# Atomic‐Scale Tailoring of Ni‐Rich Cathodes for the Development of Commercial Li‐Ion Batteries: From Laboratory to Market

**DOI:** 10.1002/advs.202515521

**Published:** 2025-11-06

**Authors:** Joonhyeon Kang, Sanghyun Lee, Young Cheol Choi, Seungjun Myeong, Sinyoung Park, Hyo Seok Kang, Jia Shin, June Woo Lee, Minkyu You, Sun Sik Shin, Yung Jong Lee, Mira Im, Jong Keun Lee, Jongpil Jegal, Kwan Soo Lee, Cheol‐Hee Park

**Affiliations:** ^1^ R&D Campus, Daejeon LG Energy Solution 188 Moonji‐ro, Yusong‐gu Daejon 34122 Republic of Korea; ^2^ R&D Campus Daejeon LG Chem 188 Moonji‐ro, Yusong‐gu Daejon 34122 Republic of Korea

**Keywords:** commercial lithium‐ion batteries, cut‐off voltages, lithium‐to‐transition‐metal input ratios, nickel‐rich cathodes, voltage profiles

## Abstract

The rapid growth of the Li‐ion battery industry has promoted the development of breakthrough materials and systems; nevertheless, achieving desired performance through sophisticated designs within well‐established systems has also become essential. Here, it is demonstrated that precise control of the lithium‐to‐transition‐metal (Li/M) ratio serves as a commercially viable lever to tune the voltage profile of Ni‐rich cathodes. This arises from the combined effects of Ni defect at Li sites (Ni_Li_) and the Ni oxidation state, as confirmed by experimental validation and density functional theory (DFT) calculations. This fundamental understanding establishes design rules for tailoring voltage profiles to meet application‐specific cut‐off voltages, thereby achieving optimal performance. This study presents a systematic framework for developing commercial cells based on atomic‐scale design principles, ensuring industrial applicability.

## Graphical abstract

The growing demand in the lithium‐ion battery industry requires the efficient development of commercial batteries that approach theoretical limits within well‐established systems. In this work, we develop a strategy for controlling the voltage profile under fixed cut‐off voltages in commercial cells by utilizing Ni‐rich cathodes. We establish an atomic‐scale design blueprint and successfully develop commercial cells tailored to the product.

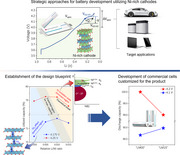

## 1 Introduction

As battery performance is considered a key specification of end products, device manufacturers and end consumers have increasingly stringent, specific, and sophisticated requirements and standards for battery suppliers.^[^ ^1^, ^2^, ^3^, ^4^ ^]^ To meet these specifications, modifications to cell design conditions and the optimization of materials—including active materials, electrolytes, binders, conductive agents, additives, and other components—are necessary.^[^ ^5^, ^6^, ^7^, ^8^ ^]^ Consequently, there is an increasing focus on exploring new materials and systems as potential candidates or enhancing conventional materials through various modifications. However, current battery manufacturers often face significant limitations when it comes to material selection and cell design due to the need to develop optimal cells under a range of tight constraints. Batteries must deliver various performance aspects required for the operation of the final product in which they are integrated.

The optimization of battery performance extends beyond the cell itself to encompass the operation of the final device, including voltage range and current requirements. Even slight improvements in energy density under such constraints can provide a decisive competitive advantage. Although some electrode materials show exceptional performance at the laboratory scale, their commercialization still faces substantial challenges and often requires long development cycles. Therefore, in today's battery industry, where demand is rapidly increasing and development timelines and costs are tightly constrained, it is essential to efficiently develop cells that meet product specifications using validated materials and established manufacturing processes. In this context, minimal adjustment of process variables offers an effective route to optimize performance while minimizing unintended changes and unnecessary trial‐and‐error during large‐scale production.

Recently, Ni‐rich (Ni > 80%) layered cathode materials have attracted significant attention due to their high practical capacity and cost‐effectiveness^[^ ^9^, ^10^, ^11^, ^12^, ^13^, ^14^, ^15^, ^16^, ^17^, ^18^, ^19^ ^]^ within the framework of validated traditional materials and systems. Understanding and utilizing phase transition behaviors, especially the H2‐H3 phase transition during charge and discharge, are crucial for maximizing their potential performance.^[^ ^20^, ^21^, ^22^, ^23^, ^24^, ^25^, ^26^, ^27^, ^28^, ^29^, ^30^, ^31^, ^32^, ^33^, ^34^ ^]^ The H2‐H3 phase transition typically creates a long voltage plateau region (V_H2‐H3_) near the charging cut‐off voltage (V_cut‐off_), and even slight optimization of the relative position of these voltages can result in a capacity difference of several percent.

In this study, we propose a practical and effective methodology for tailoring Ni‐rich cathode materials to specific products and operational conditions using the lithium‐to‐metal (Li/M) ratio (M═Ni, Co, Mn) as a single process variable. We show that precise atomic‐level control, achieved by adjusting precursor input ratios and gradually controlling anti‐site defects (Ni_Li_) linked to the H2‐H3 transition, realizes the desired performance in commercial‐scale batteries. Previous studies have primarily described structural and basic electrochemical changes resulting from Li/M variation;^[^ ^35^, ^36^, ^37^, ^38^, ^39^, ^40^, ^41^ ^]^ we apply these findings practically to design batteries with voltage profiles tailored to specific cut‐off voltages.

Furthermore, by investigating the competition between the effects of Ni_Li_ defects and stoichiometric redox capability depending on Li/M variation, we have developed a 2D design blueprint capable of accommodating a range of cut‐off voltages, successfully applied in commercial cell design. We use density functional theory (DFT) calculations to predict the effects of Ni_Li_ and closely examine their behavior. The relationship between intrinsic defects and the key process variable (Li/M ratio) is elucidated using analytical techniques such as X‐ray diffraction (XRD) and the superconducting quantum interference device (SQUID). Building on these fundamental principles, our ultimate goal is to efficiently develop commercial cells by utilizing the Li/M ratio as a single, fundamental design factor, thereby customizing voltage profiles to be optimized for achieving the desired specifications.

## 2 Results and Discussion

### 2.1 Strategies for Optimizing the Capacity of Ni‐Rich Cathodes

**Figure 1:**
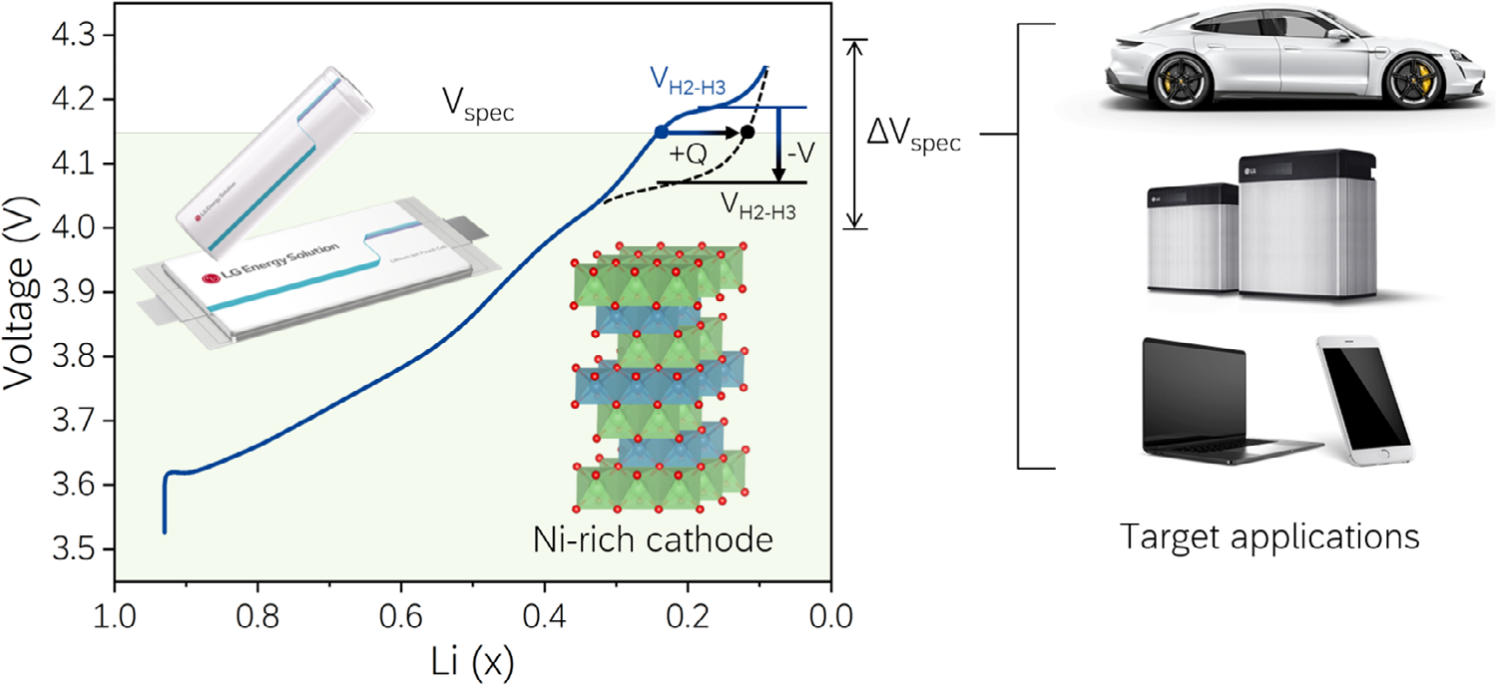
Strategies for optimizing capacity within voltage limits (V_spec_) specified in various applications utilizing Ni‐rich cathodes (the provided product images are not related to this study).

Battery manufacturers optimize battery designs by using appropriate materials to meet the requirements of various customers; they start with a clear concept from the material stage to enable battery development with the desired performance. The optimization strategy of the battery capacity using Ni‐rich cathode materials is shown in **Figure** **1**. The voltage profile exhibits a distinctive plateau arising from the Ni‐rich cathode materials at charge termination (V_H2‐H3_)_,_ resulting in a significant capacity output in the region. Therefore, the location and extent of the region within the voltage range play important roles in determining the final battery performance.

As the voltage limit is often determined in the subtle voltage range near the V_H2‐H3_ of Ni‐rich cathodes based on specific products and the application requirements of each customer, proper control of the voltage plateau (V_H2‐H3_) is a key aspect in utilizing Ni‐rich cathode materials. Conversely, if V_H2‐H3_ can be controlled via minimal modifications of the other aspects, efficient development of batteries with a wide range of performances and applications is feasible, bypassing the requirement for extensive modifications such as the application of new active materials or significant changes to the cell design.

The available capacity is ultimately determined from the relative position of the adjusted V_H2‐H3_ and cut‐off voltage. When the cut‐off voltage is within the H2–H3 transition range, the capacity utilized in this range dominates the overall capacity. However, when the cut‐off voltage is far beyond the aforementioned range, the capacity is determined simply by the concentration of redox centers that extract electrons up to that voltage, depending on the oxidation state and concentration of redox‐active transition metals. Therefore, designing materials that match the utilization voltage range is essential, and this will be demonstrated through two characteristic cut‐off voltages discussed later.

**Figure 2:**
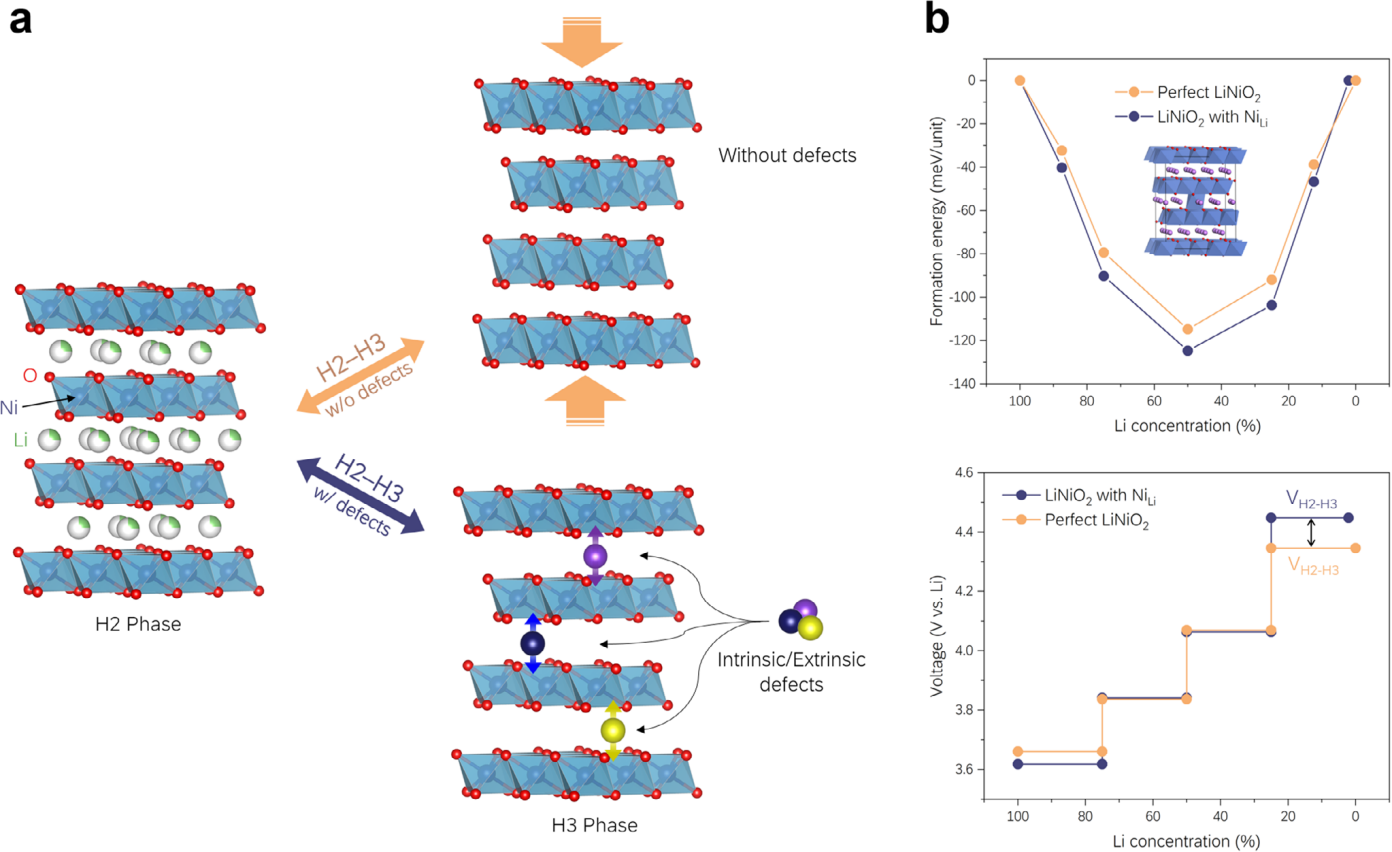
Voltage modulation through atomic‐level modification of H2–H3 phase transition. a) Schematic of the role of intrinsic/extrinsic defects during H2–H3 phase transition. b) DFT calculation results for changes in formation energy and resulting voltage profiles relative to Li concentration in LiNiO_2_, with and without Ni_Li_ defects. The H2–H3 phase transition range for LiNiO_2_ with the Ni_Li_ model is reduced by 1/48 (≈2.1%) of the Li concentration, which corresponds to the atomic ratio of Ni_Li_ defects in the model.

As mentioned earlier, the H2–H3 phase transition and voltage plateau are signature properties of Ni‐rich cathode materials. During charge termination, the extraction of Li from a dilute Li layer within the crystal structure of Ni‐rich materials induces a decrease in negative charge and repulsion of oxygen between transition metal slabs (TMO_6_).^[^ ^42^, ^43^ ^]^ This leads to a rapid collapse of the Li layer, as depicted in **Figure** **2**a. To control its collapse, the incorporation of immobile intrinsic/extrinsic defects into the Li layer has been considered promising for altering the phase transition behavior.^[^ ^43^, ^44^, ^45^, ^46^, ^47^ ^]^ The effect of such defects is known as the “pillar effect”, which is typically considered to prevent the collapse of the Li layer.^[^ ^44^ ^]^

In this study, we focus on designing batteries with the desired specifications by appropriately controlling such defects. From DFT calculations on a LiNiO_2_ model, the variations in V_H2‐H3_ due to the presence of various intrinsic defects in Ni‐rich cathode materials were predicted. As shown in Figure 2b, the presence of Ni_Li_ increases V_H2‐H3_ due to changes in the formation energy depending on the Li composition. Therefore, material synthesis is focused on increasing Ni_Li_ to increase V_H2‐H3_, whereas the opposite is pursued to decrease V_H2‐H3_.

To control such defects, the Li/M ratio was considered a promising process variable, representing the molar ratio of Li to transition metals based on the input amount. Generally, the Li/M ratio is used as a process variable to ensure stoichiometry in chemical reactions, and its precise control is crucial. Depending on the synthetic conditions, deviations from the stoichiometry can significantly affect electrochemical properties.^[^ ^38^, ^39^, ^40^, ^41^ ^]^ Additionally, a Li‐rich or Li‐poor environment that favors the generation of specific defects can be established during synthesis by adjusting the Li/M ratio. Based on theoretical calculations of the Ni_Li_ defect formation energy in Li‐rich and Li‐poor environments, we anticipate that an increase in the Li/Ni ratio can induce a decrease in Ni_Li_ (Figure S3, Supporting Information). Although other process variables, including the sintering temperature and transition metal composition, can be adjusted, they induce major unintended changes in the particle size and voltage profile, thereby deviating from our objective of specifically controlling V_H2‐H3_ while minimizing its impact on other parameters.

Furthermore, by adjusting the Li/M ratio using the same precursor and optimizing the feed ratio according to specific applications, significant advantages can be achieved in terms of process efficiency, convenience, cost‐effectiveness, and quality management. Considering business aspects and theoretical backgrounds, in this study, we synthesize Ni‐rich cathode materials with controlled Li/M ratios and investigate their structural and electrochemical characteristics.

### 2.2 Synthesis and Characterization of Defect‐Controlled Ni‐Rich Cathode Materials

In this study, we used a slightly higher input Li/M ratio (M═Ni, Co, Mn), typically greater than 1, depending on the type or purpose of the cathode material. To validate our design concept, we synthesized laboratory‐scale Ni‐rich NCM by adjusting the Li/M input ratio at regular intervals of 0.01 from a specific value R_0_. For convenience, the difference between the adjusted Li/M input ratio and R_0_ is referred to as the relative Li/M ratio. The transition metal ratio was fixed at a specific value, with a Ni:(Co+Mn) ratio of 9:1.

To describe the synthesized samples, we considered six crystal model candidates: stoichiometric [Li]_3a_[Ni]_3b_[O_2_]_6c_ (Model 0), stoichiometric Li/Ni cation mixing [Li_1‐x_Ni_x_]_3a_[Ni_1‐x_Li_x_]_3b_[O_2_]_6c_ (Model 1), non‐stoichiometric Li deficiency [Li_1‐x_]_3a_[Ni]_3b_[O_2_]_6c_ (Model 2), non‐stoichiometric Ni excess [Li_1‐x_Ni_x_]_3a_[Ni]_3b_[O_2_]_6c_ (Model 3), non‐stoichiometric Li excess [Li]_3a_[Ni_1‐_ *_x_* Li*_x_*]_3b_[O_2_]_6c_ (Model 4), and non‐stoichiometric O deficiency [Li]_3a_[Ni]_3b_[O_2‐x_]_6c_ (Model 5).

Variations in the Li/M ratio within each crystal model lead to distinct changes in the structural and magnetic properties. Therefore, we simplified our samples using XRD and SQUID analyses to the [Li_1‐x_Ni_x_]_3a_[Ni_1‐y_Li_y_]_3b_[O_2_]_6c_ formula, which includes Models 1, 3, and 4. Moreover, the Rietveld refinement results indicated that all samples exhibited a distinctive feature of finite Ni_Li_ defects and negligible levels of Li_Ni_ defects (y≈0) (a detailed result is provided in Figure S4 and Table S1, Supporting Information). Therefore, Model 3 was selected as a representative model to describe the samples under our synthetic conditions (a detailed discussion is provided in Note S1, Supporting Information).

**Figure 3:**
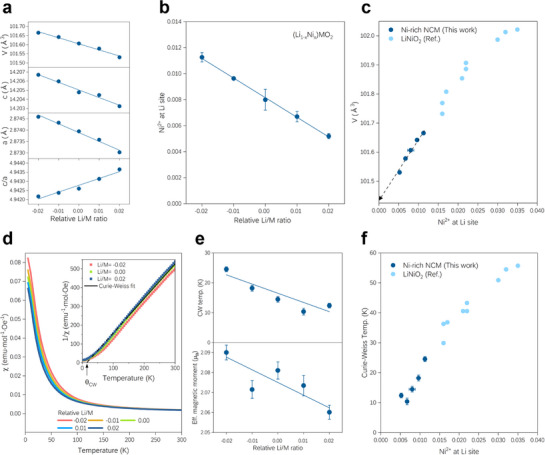
Structural and magnetic properties of Ni‐rich cathode materials depending on the Li/M ratio. a) Unit cell parameters and b) Ni occupation at Li sites (Wyckoff position 3a) obtained from Rietveld refinement. Representative XRD patterns and the Rietveld refinement results are presented in Figure S4 (Supporting Information). c) Relationship between unit cell volume (*V*) and Ni at the Li site. As Ni_Li_ decreases, the stoichiometric NCM unit‐cell volume is approached. d) Zero‐field cool magnetic susceptibility under 1 T and inverse magnetic susceptibility with Curie–Weiss fitting (inset). e) Curie–Weiss temperature and effective magnetic moment obtained through fitting. f) Correlation between Ni at the Li site and Curie–Weiss temperature. As the Ni_Li_ defect decreases, the Curie–Weiss temperature approaches ≈0 K. The error bars for the refined lattice constants and magnetic susceptibility are very small to be visible compared to the size of the symbols. Data in Figure 3c,f are adapted from Table 1 and Table 2 in Ref. [37]. Copyright The Electrochemical Society. Adapted by permission of IOP Publishing. All rights reserved. https://doi.org/10.1149/1945‐7111/ac33e5.

As shown in **Figure** **3**a,b, an increase in the relative Li/M ratio reduces the occupancy of Ni^2+^ at the Li sites, which decreases the lattice parameters *a*, *c*, and unit cell volume *V*. When the amount of Ni^2+^ (x) at the 3a site is replaced by Li^+^ (R_Ni2+_ = 0.69 Å → R_Li+_ = 0.76 Å), the charge neutrality induces an increase in the Ni oxidation state at the 3b site from +3−x to +3 (R_Ni2+_ = 0.69 Å → R_Ni3+_ = 0.56 Å) (Table S2 and Figure S5, Supporting Information). Oxidation reduces the ionic radius of Ni and outweighs the effect of Ni^2+^ being replaced by Li^+^, leading to a decrease in *a*, *c*, and *V*. This is also consistent with the trend of lattice parameter variation calculated from DFT (Table S3, Supporting Information). For convenience, the layered structure can be described as a distorted cubic structure.^[^ ^21^, ^48^, ^49^ ^]^ In the rock‐salt structure, where layers of Li and transition metals are not distinctly separated but are randomly mixed, the arrangement in the [111] direction aligns with the *c*‐axis direction in the layered structure. Due to the alternation of Li and Ni cation ordering along the [111]_c_ direction in the ideal cubic rock‐salt structure, the *c/a* ratio deviates from the ideal cubic ratio of *c_o_/a_o_* = 2√6. The reduction in Ni_Li_ defects causes an increase in the average ionic‐size difference between the 3a and 3b sites, along with rhombohedral distortion that increases the *c/a* ratio.

Furthermore, we verified the structural analysis consistency by plotting the Ni_Li_ defects and *V* against the Li_1‐x_Ni_1+x_O_2_ reference,^[^ ^37^ ^]^ as shown in Figure 3c. The crystal structure of Ni‐rich NCM is similar to that of Li_1‐x_Ni_1+x_O_2_ in terms of *V* and Ni_Li_ defects. The linear relationship between the Ni_Li_ defect and *V* observed in this study aligns with that observed in Li_1‐x_Ni_1+x_O_2_. Thus, our samples approach the line from the region with a large number of Ni_Li_ defects (> 1.5%) to that with a small number of defects (< 1.5%) as the Li/M ratio increases. However, an ideal stoichiometric Ni‐rich NCM with free‐Ni_Li_ defects could not be achieved. According to the guidelines, we expect the ideal *V* and the *I*_003_/*I*_104_ ratio to be ≈101.45 Å^3^ and 1.315, respectively (Figure S6, Supporting Information).

A SQUID magnetometer was used to investigate the Ni oxidation state and presence of Ni_Li_ or Li_Ni_ defects, which are correlated to the effective magnetic moment and Curie–Weiss temperature, respectively (Figure 3d,e). With an increase in the Li/M ratio, Ni^2+^ (S = 1) is gradually replaced by non‐magnetic Li^+^ (S = 0) at the 3a site, leading to a reduction in the average effective magnetic moments of the samples. In the ideal LiNiO_2_ structure, an in‐plane exchange interaction (*J*) occurs through the edge‐sharing octahedra on the triangular transition metal lattice, while an out‐of‐plane exchange interaction (*J_op_*) occurs between the triangular transition metal layers (Figure S7, Supporting Information). When a magnetic Ni^2+^ is present at the Li site (3a), an additional *J_op_* ' interaction occurs. Subsequently, a decrease in the amount of Ni_Li_ causes a reduction in the *J_op_* ' exchange interaction, resulting in a lower Curie–Weiss temperature, which is proportional to the average exchange interaction (as discussed in Note S2, Supporting Information). The changes in the effective magnetic moment and Curie–Weiss temperature as a function of the Li/M ratio are shown in Figure 3e.

The decreasing trend of the Curie–Weiss temperature with Ni_Li_ defects and the literature values for Li_1‐x_Ni_1+x_O_2_ are shown in Figure 3f. Although NCM is generally more complicated than that of Li_1‐x_Ni_1+x_O_2_ and includes a mixture of Ni, Co, and Mn, our Ni‐rich NCM follows the trend of Li_1‐x_Ni_1+x_O_2_. Thus, the Ni_Li_ defects can be indirectly regulated with high precision and effectiveness by controlling the Li/M input ratio, as demonstrated by the changes in the structural and magnetic properties.

While techniques such as neutron diffraction and XANES could, in principle, provide additional or more direct validation, in our samples with finely tuned Li/M ratios, variations in Ni_Li_ defects and oxidation state are indirectly yet more sensitively reflected in lattice parameter changes and magnetic properties. The combined results from XRD, SQUID, and DFT already offer robust evidence for a decrease in Ni_Li_ defects and an increase in the Ni oxidation state with increasing Li/M ratio. Thus, complementary probes in a broader range of Li/M tuning would further confirm our observations, and the present findings are in good agreement with previous reports on Ni‐rich layered oxides.^[^ ^37^, ^38^, ^39^, ^40^, ^41^ ^]^

**Figure 4:**
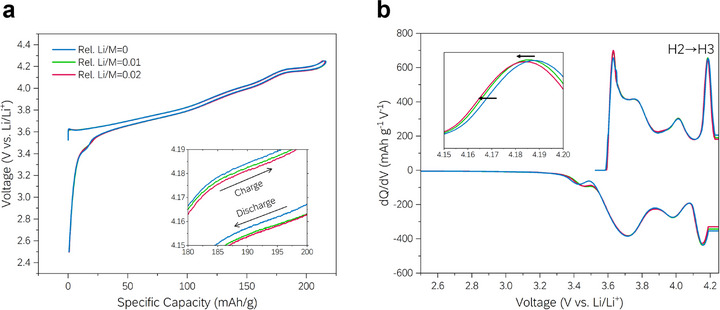
Electrochemical properties of Ni‐rich cathode materials with controlled Li/M ratios. a) Voltage profiles for different Li/M ratios in the range of 2.5–4.25 V. The inset shows an enlarged graph of the plateau region observed during H2–H3 phase transition. b) Corresponding dQ/dV curves for each voltage profile. Each curve has been smoothed for better understanding, and the inset is enlarged to compare the peak during H2–H3 phase transition.

To investigate the correlation between the structural and electrochemical properties, materials synthesized with different relative Li/M ratios (0, 0.01, and 0.02) were tested using a coin half‐cell (**Figure** **4**). As illustrated in the voltage profiles in Figure 4a, the H2–H3 plateau (V_H2–H3_) systematically shifts to lower voltages with increasing Li/M ratio during both charge and discharge, indicating that the voltage evolution is governed by thermodynamic rather than kinetic effects. This interpretation is further supported by the dQ/dV analysis in Figure 4b, where both charge and discharge peaks shift in parallel while the hysteresis (Δ*V*) remains nearly constant at ≈0.030 V (Table S4 and Figure S8, Supporting Information). Importantly, the observed trends agree well with the thermodynamic shifts predicted by our DFT calculations, reinforcing that adjusting the Li/M input ratio effectively modulates V_H2–H3_ in Ni‐rich cathodes. Thus, defect‐controlled materials are successfully synthesized according to the design concept, as confirmed by structural and magnetic analyses, affording the expected electrochemical properties.

### 2.3 Large‐Scale Production and Application to Commercial Cells

Based on the validation of the experimental results in accordance with the laboratory design, we manufactured large‐scale Ni‐rich cathode materials for commercial‐grade cells in subsequent phases. The synthesized products were classified into specific grades (LM00, LM05, LM10, and LM15) based on a predetermined Li/M input ratio (R_0_) at the laboratory scale, with corresponding increments of 0.005, 0.01, and 0.015, as listed in Table S5 (Supporting Information).

**Figure 5:**
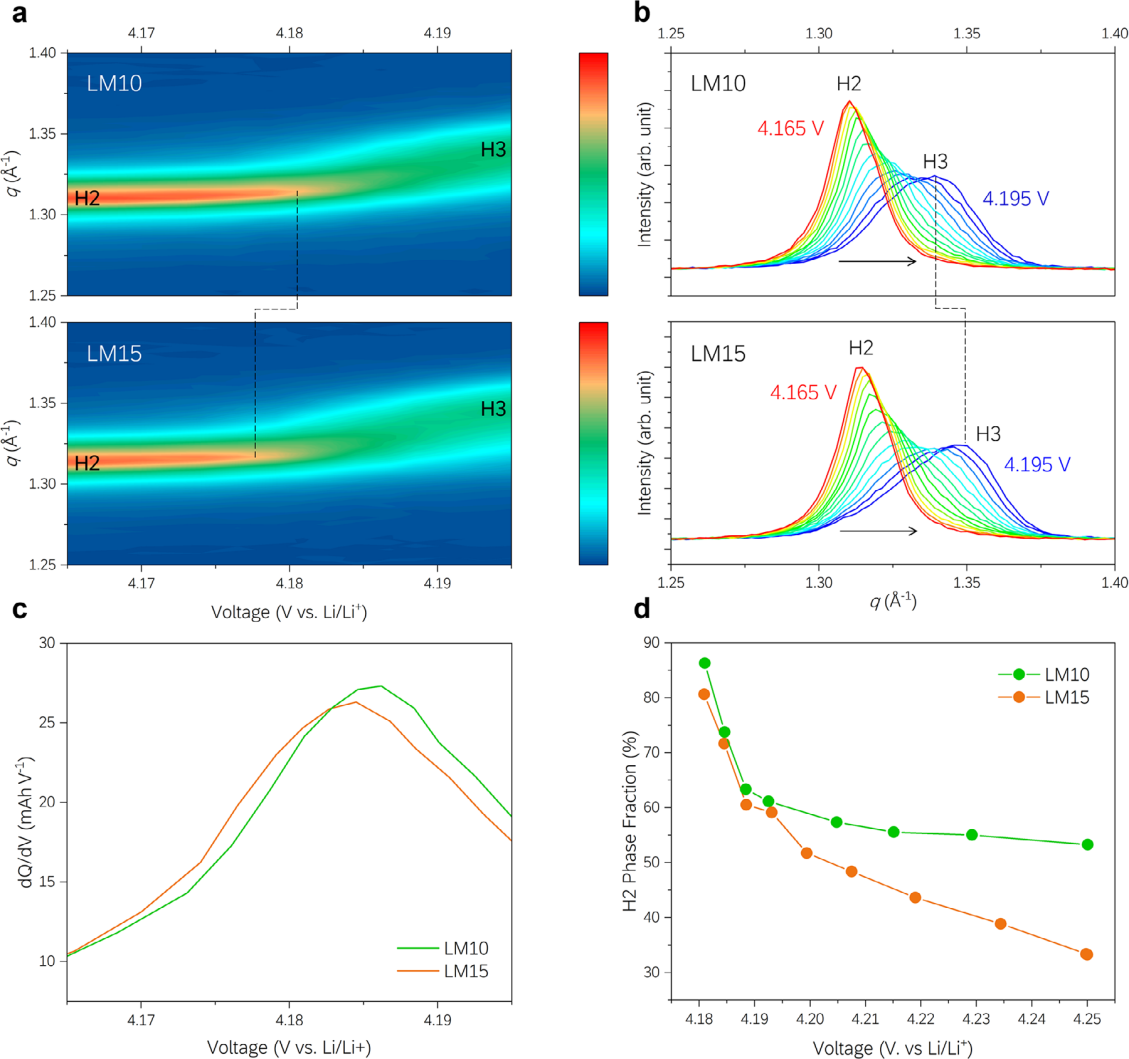
*Operando* XRD analysis results for LM10 and LM15. a) Contour plot of the (003) peak changes in the ≈4.165–4.195 V range during charging. b) Individual XRD patterns showing the phase‐transition behavior. c) Comparison of dQ/dV peaks for the H2–H3 transition. d) Variation in H2 phase fraction during charging obtained from fitting the area with two peaks in the XRD pattern during charging.

To directly observe the H2–H3 phase transition behavior, we fabricated coin half‐cells using the aforementioned grade products and performed *operando* XRD analysis while charging them to 4.25 V. Comparisons between LM10 and LM15 (**Figure** **5**) revealed that LM15, with a higher Li/M ratio, exhibits a gradual decrease in the H2 phase intensity at lower voltages, as identified by the (003) peak shown in Figure 5a. The XRD patterns in the same voltage range are shown in Figure 5b, where LM15 exhibits a significant transition at 4.195 V. This results in lower and higher proportions of the H2 and H3 phases, respectively, with a greater peak shift toward a high scattering vector (*q* = 4πsin θ/λ). As the H3 phase is characterized by the rapid collapse of the *c*‐axis, the changes in the peak position and shape, including a shift toward high *q*, reflect the relative dominance of the H3 phase. Such an observation is further confirmed by the dQ/dV profile (Figure 5c) and the variation in the H2 phase fraction, which are obtained through two‐phase fitting of the (003) peak and calculated as the integrated intensity ratio *I*_H2_/(*I*_H2_+*I*_H3_) (Figure 5d). The evolution of other major reflections, along with the extracted lattice parameters, is provided in the Supporting Information (Figures S9 and S10, Supporting Information). Thus, the electrochemical and structural characteristics observed via laboratory‐scale synthesis demonstrate consistent trends and are applicable to large‐scale production.

**Figure 6:**
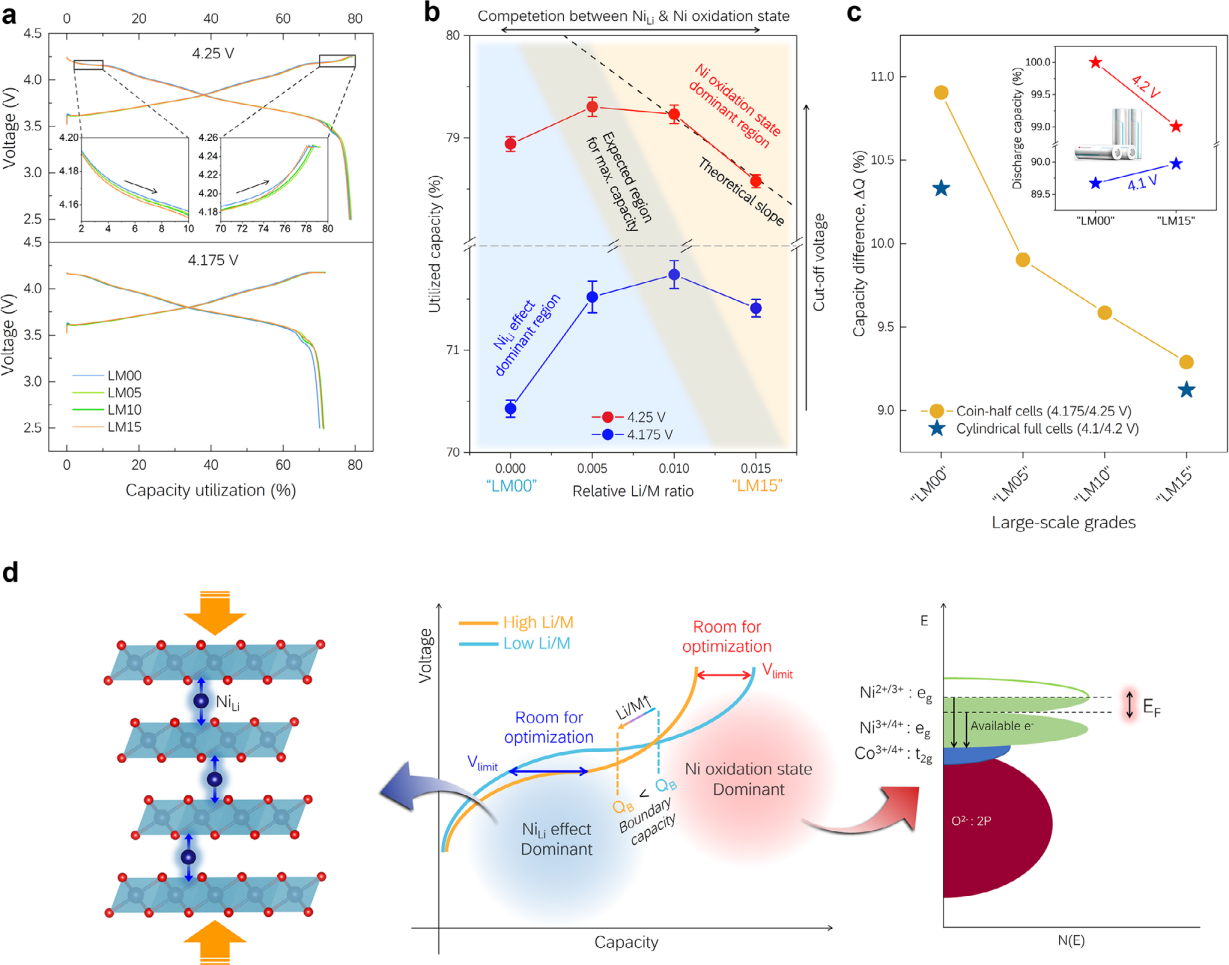
Development of commercial cells based on the design schematic of Ni‐rich cathodes. a) Initial voltage profiles at the conditions of cut‐off voltage: 4.25 V (top) and 4.175 V (bottom), obtained through coin half‐cell tests for each grade. Capacity is shown as a relative value to the theoretical capacity of stoichiometric Ni‐rich NCM. b) Capacity changes as a function of relative Li/M ratios at cut‐off voltages of 4.175 and 4.25 V. The dashed line, corresponding to Ni_ox_(E) = 3.82 and Co_ox_(E) = 3.48 based on our calculations, is included for comparison of slope with the actual capacity. c) Capacity difference between high and low cut‐off voltages for coin half‐ and cylindrical full‐cells using large‐scale Ni‐rich cathode materials: ΔQ=(Q_4.25V_‐Q_4.175 V_)/Q_4.25 V_ for coin half‐cells and ΔQ=(Q_4.2V_‐Q_4.1 V_)/Q_4.2 V_ for cylindrical full‐cells. The capacity of the cylindrical cell is based on the first discharge capacity for 4.2 V and the consecutive second discharge capacity for 4.1 V, after the activation process, as shown in the inset (the capacity is expressed as a relative value when the capacity of LM00 at 4.2 V is set to 100%). d) Schematic of the origin of capacity behavior changes with Li/M variations under cut‐off voltage conditions inside and outside the H2–H3 phase transition region.

Further, we evaluated the electrochemical characteristics depending on the cut‐off voltage for each grade using coin half‐cells to obtain atomic‐level design schematics for commercial cylindrical‐cell manufacturing. The cut‐off voltages were selected as two specific voltages of interest within the key voltage range based on the position of the dQ/dV peaks in Figure 4b: The voltages i) during the H2–H3 phase transition (4.175 V) and ii) at the near‐complete state (4.25 V). The evaluation results for each voltage and grade are presented in **Figure** **6**a as electrochemical profiles. The capacity is expressed as a ratio relative to the theoretical capacity of the stoichiometric composition. In the enlarged region of the voltage profile with a cut‐off voltage of 4.25 V, the H2–H3 plateau is well‐controlled according to the designed Li/M ratio. Furthermore, in the voltage profile at a cut‐off voltage of 4.175 V, the capacity of LM00 with the lowest Li/M ratio is notably lower in the discharge.

The average capacity values obtained from four different tests with different cut‐off voltages relative to the Li/M ratio employed in the design are shown in Figure 6b (charge capacity) and Figure S11 (Supporting Information) (discharge capacity). Evidently, the capacity initially increases with the Li/M ratio at both 4.175 and 4.25 V, reaching an optimal value before decreasing (Figure 6b). The observed trend is attributed to a capacity enhancement due to the decrease in Ni_Li_ and V_H2‐H3_ with an increase in the Li/M ratio, followed by a decrease in capacity owing to other influencing factors. Additionally, a more significant decrease in the capacity is observed at a higher cut‐off voltage of 4.25 V, indicating the more pronounced effect of factors causing capacity reduction at higher cut‐off voltages. A similar trend is observed for the discharge capacity, as shown in Figure S6 (Supporting Information).

The oxidation state of Ni tends to increase with increasing Li/M ratio, which is corroborated both by simple stoichiometric estimations (Figure S5, Supporting Information) and experimentally through BVS calculations (Figure S12, Supporting Information). The maximum oxidation state of Ni is limited to +4 under battery operating conditions; therefore, an increase in the initial oxidation state of Ni results in a decrease in the number of electrons that can be utilized by Ni.^[^ ^50^, ^51^, ^52^ ^]^ Similarly, when the average oxidation state of Ni is determined at a specific voltage, the number of electrons that can be utilized by Ni is determined by the initial oxidation state of Ni. Then, the available charge from Ni to the potential *E* can be calculated using Equation (1).

(1) $$\mathrm{Available}\;\mathrm{charge}=\mathrm{Ni}\;\mathrm{concentration}\;\times \;(Ni_{\mathrm{ox}}\left(E\right){-\mathrm{Ni}}_{\mathrm{ox}}^{\mathrm{Initial}}\;)$$

where *Ni_ox_* (*E*) represents the oxidation state of Ni at a potential *E*. The same equation is used to consider the charge available from Co. Assuming that the initial oxidation states of Co and Mn are fixed at +3 and +4, respectively, the concentrations of Ni and Co within the structure and the initial oxidation state of Ni are expressed as functions of the Li/M ratio (further details in Note S3, Supporting Information). Thus, the available charge or capacity is approximated asymptotically by a linear relationship for the Li/M ratio values within the range of interest (Figure S13, Supporting Information), as represented by the dashed line in Figure 6b. At 4.25 V, the capacity decreases to a level comparable to the theoretically calculated slope for relative Li/M ratios between 0.01 and 0.015, indicating that the range is within the region dominated by the oxidation state of Ni, which depends on the initial stoichiometry.

At an upper cut‐off voltage of 4.25 V, where the H2–H3 phase transition is nearly complete, the capacity advantage gained by initiating the H2–H3 transition at lower voltages through an increase in the Li/M ratio diminishes. Instead, the predominant effect is the capacity loss caused by the increase in the initial oxidation state of Ni. However, when the upper cut‐off voltage is positioned within the H2–H3 transition range (at 4.175 V), the achievable capacity until the attainment of the cut‐off voltage varies significantly depending on how early the plateau forms at lower voltages. Therefore, the effect of adjusting V_H2‐H3_ through Ni_Li_ becomes more pronounced across a wider range of Li/M ratios, as observed in Figure 6b. An extensive region of increasing capacity with the Li/M ratio at 4.175 V is observed, while a substantial decrease in the capacity is observed at 4.25 V. Notably, significant capacity changes occur as the relative Li/M ratio increases from 0 to 0.005 at 4.175 V and from 0.010 to 0.015 at 4.25 V.

When the cut‐off voltage is increased beyond 4.25 V, thereby approaching the theoretical capacity, it is predicted that the capacity will continue to decrease as the Li/M ratio increases, based on calculations without considering the voltage plateau (Figure S13, Supporting Information). Therefore, we conclude that the tendency of the capacity to decrease upon an increase in the Li/M ratio becomes stronger as the cut‐off voltage increases. As a result, as the capacity utilization increases, the Li/M range dominated by Ni_Li_ effects becomes narrower, whereas the range dominated by the Ni oxidation state becomes wider. In other words, as we try to utilize the capacity closer to the theoretical limit, the tunability by voltage decreases and becomes dependent on the initial stoichiometry.

Furthermore, the initial oxidation state of Ni increases with the Li/M ratio, while the Ni concentration decreases, causing a decrease in capacity at the voltage where the H2–H3 transition ends. Therefore, the region dominated by the oxidation state of Ni is attained from lower capacity values at higher Li/M ratios, such that a border is formed at a lower position. Based on the observed trends, we approximately indicate the regions where each effect has a dominant impact and the composition range that is expected to afford the maximum capacity as the cut‐off voltage and an increased utilized capacity. A similar trend is observed for the discharge capacity, as shown in Figure S11 (Supporting Information).

Using the atomic‐scale design blueprint shown in Figure 6b, we successfully manufactured commercial cylindrical cells. The cylindrical cells were produced using LM00 and LM15 for high and low cut‐off voltages, respectively, and the fundamental principles operated as intended. As shown in the inset of Figure 6c, the capacity of cells using LM15 and LM00 is superior at cut‐off voltages of 4.1 and 4.2 V, respectively. The voltages have comparable levels of positive electrode potential at 4.175 and 4.25 V for full cells using graphite as the negative electrode and coin half‐cells, respectively. Although the evaluated C‐rate is higher (0.5 C vs 0.2 C), the results suggest that thermodynamic factors could play a dominant role, provided that the overpotentials remain comparable across different grades (Table S4, Supporting Information), with the constant‐voltage step at the end of charge further mitigating kinetic effects.

LM00 and LM15, designed to achieve volumetric energy densities up to ≈750 Wh L^−1^, were further subjected to extended evaluations. As summarized in Tables S7 and S8 and Figure S14 (Supporting Information), both grades exhibited comparable performance in long‐term cycling, high‐temperature storage, and gas evolution, all within acceptable levels, despite the structural effects caused by Ni in the Li layer (Table S9, Supporting Information). Moreover, they successfully passed all safety evaluations required for product qualification, including mechanical and thermal abuse tests, thereby confirming their industrial feasibility. Additional results in Figure S15 and Table S10 (Supporting Information) further confirm that all investigated grades (LM00–LM15) maintain excellent energy retention, supporting the general applicability of this design approach.

Additionally, the use of higher Li/M ratios leads to a reduction in the capacity difference between the conditions of high and low cut‐off voltages (Figure 6c). Such a phenomenon is observed due to the opposing dominant effects of the Ni oxidation state and Ni_Li_ effect at high and low cut‐off voltages, respectively, resulting in compensatory capacity variations. Comparisons between the 4.175 and 4.25 V profiles of the coin half‐cell reveal that the observed trend is applicable to the commercial cylindrical full cell at 4.1 and 4.2 V, respectively. As an essential specification criterion for battery suppliers and customers, the cut‐off voltage is positioned within a subtle range in the Ni‐rich cathode, where it is influenced by V_H2‐H3_, giving rise to the observed interesting phenomena.

The capacity variation behavior during the change in the cut‐off voltage is presented in Figure 6d. First, when the cut‐off voltage is within the H2–H3 transition range, the dominant effect of Ni_Li_ that allows the control of V_H2‐H3_ results in an increase in capacity with the Li/M ratio. However, when the cut‐off voltage is set in the range after phase transition and the voltage plateau has already passed, the available number of electrons is limited to a specific voltage by the initial concentration and oxidation state of Ni (where the latter plays a more significant role), thereby determining the overall capacity. Thus, by adjusting the process parameters of the Li/M ratio and designing a voltage profile tailored to each specified cut‐off voltage, we conveniently develop commercial‐level batteries with optimal capacities, thereby narrowing the gap between the achievable and theoretical capacities of each product.

While our study mainly focuses on the thermodynamic effects of varying Li/M ratios and does not incorporate detailed kinetic analyses such as EIS or GITT, such investigations could serve as a highly interesting research topic—particularly for optimizing cathode performance under different power conditions—when integrated with our framework. Nevertheless, within the scope of the present work, the approach demonstrates effectiveness in achieving high performance, reliable quality control, and efficient product development simply by adjusting the input ratio of the same precursor.

## 3 Conclusion

In summary, we have developed commercial Li‐ion batteries with desired performances through sophisticated design tailored to each operating voltage at the atomic scale. For Ni‐rich cathodes, the available capacity is greatly influenced by the relative position of their voltage plateau (V_H2‐H3_) and the upper voltage limit. Therefore, we established a design strategy to control the H2‐H3 transition within the given voltage range specified for each product. Using DFT calculations, the Li/M ratio was derived as an effective process variable for implementing the strategy. After the laboratory‐scale preparation of samples with controlled Li/M ratios, the design concept was validated by analyzing their structural and electrochemical properties. Through the evaluation of mass‐produced materials, we finalized the design blueprint and successfully developed commercial cylindrical cells that meet the required specifications. Our work from the laboratory to the market highlights the vital role of a well‐defined design in the early stages of battery development. Our approach exponentially increases the efficiency of new product development and is also applicable to solving quality‐related issues. The proposed methodology has a wide range of applications, enabling the fine‐tuning of each material depending on battery operating conditions. More broadly, this strategy establishes a generalizable framework that bridges atomic‐scale design principles with practical realization, providing universal guidance for diverse battery chemistries and applications.

## 4 Experimental Section

### DFT Calculations

All DFT calculations were performed using the Vienna ab initio simulation package (VASP) and the projected augmented wave (PAW) method.^[^ ^53^, ^54^, ^55^ ^]^ The Perdew–Burke–Ernzerhof (PBE) functional was used to treat exchange and correlation, and the GGA+U method was adapted to describe the *d* electron of Ni with a Hubbard U parameter of 6.2 eV for Ni.^[^ ^56^, ^57^ ^]^ A kinetic energy cut‐off of 520 eV and a gamma‐centered 2×2×1 *k*‐point sampling were applied. The structures were optimized until the forces on the atoms were < 0.02 eV Å^−1^ with cell relaxation. A hexagonal 4×4×1 supercell structure with rhombohedral symmetry (*R* $\bar{3}$ *m* space group) was employed to perform perfect LiNiO_2_ model (Li_48_Ni_48_O_96_) calculations. To construct LiNiO_2_ using the Ni_Li_ model (Li_47_Ni_49_O_96_), one of the 48 Li atoms was substituted with a Ni atom (Ni_Li_ defect ratio = 1/48). Therefore, the range of the H2–H3 phase transition for the voltage profile of LiNiO_2_ with the Ni_Li_ model was shortened by 1/48 of the Li concentration (Figure 2b).

### Sample Preparation

The laboratory‐scale powder sample was synthesized via a solid‐state reaction,^[^ ^21^, ^37^ ^]^ with varying Li/M ratios in small steps of 0.01 near the specific R_0_ value, which was controlled by the input ratio of the LiOH·H_2_O and M(OH)_2_ (M═Ni, Co, Mn) precursor. The transition metal ratio was fixed at a specific value, with a Ni:(Co+Mn) ratio of 9:1. The mixture of LiOH·H_2_O and M(OH)_2_ was calcined at temperatures between 600–900 °C in an O_2_ atmosphere. Mass‐produced grades were manufactured by scaling up from the laboratory scale (at the g level) to the ton level using mass‐production equipment. Detailed synthetic conditions for this study were confidential and cannot be disclosed, even between the cathode material and battery manufacturers. Nonetheless, the experimental concept is simple and can be implemented by systematically adjusting the Li/M ratio, as previously mentioned. Therefore, this study can be reproduced regardless of the synthetic conditions, and the proposed principle is generally applicable.

### Powder X‐Ray Diffraction

X‐ray powder diffraction was conducted in Bragg–Brentano reflection geometry using a Bruker D8 Endeavor X‐ray diffractometer (Cu target), while Rietveld analysis was conducted using TOPAS Rietveld software.^[^ ^58^ ^]^ Owing to the small step size of the Li/M input ratio, XRD and Rietveld analyses were conducted carefully. First, the wide 2θ range of 10–125° was measured with a small step of Δ2θ = 0.01° to obtain as many *hkl* peaks as possible and increase the precision of the lattice constant. To minimize the sampling error (powder surface roughness and sample displacement), instrumental measurement error, and Rietveld calculation error, five independent powder samples were prepared for the same Li/M ratios while carefully monitoring the surface of the powder sample. In Rietveld analysis, a fundamental parameter approach using the double‐Voigt method (isotropic crystalline size and micro‐strain)^[^ ^59^ ^]^ was employed. Rietveld analysis provided each powder sample with an acceptable goodness of fit, with R_wp_ values ranging from 5 to 6% and small differences in the curve from low to high 2θ angle regions. The chemical structures were visualized using VESTA software.^[^ ^60^ ^]^

### SQUID

A SQUID‐VSM magnetometer (Quantum Design, MPMS‐7) was used to measure the zero‐field cool magnetic susceptibility from 5 to 300 K under an applied magnetic field of 1 T at the Korea Basic Science Institute (KBSI). Curie–Weiss fitting was applied to the inverse magnetic susceptibility from 150 to 300 K to obtain the Curie–Weiss temperature (θ_CW_) and effective magnetic moment (µ_eff_).

### Quantitative Analysis of Cathode Materials (ICP)

ICP‐OES (Perkin Elmer) was used to determine the Li/M ratios of the synthesized Ni‐rich cathode materials. The samples were prepared via acid digestion using hydrochloric acid and hydrogen peroxide. After digestion, the diluted liquid samples were introduced through a peristaltic pump and a Scott‐type chamber with a Mira Mist nebulizer. The emission lines of Li (670.784 nm), Ni (231.604 nm), Co (228.616 nm), and Mn (257.610 nm) were quantified via radial plasma viewing.

### Cell Preparation and Electrochemical Tests—Coin Half‐Cells

All coin half‐cells were manufactured following the same method and measurement conditions, excluding the ones with active material cathodes. First, a slurry was prepared by mixing the synthesized NCM active material, conductive agent (Super C), and binder (PVDF) in an N‐methylpyrrolidone (NMP) solvent to manufacture a cathode. Second, the slurry was coated on one side of an aluminum collector, which was then dried and roll‐pressed at 130 °C to produce the electrode. A Li metal electrode was used as the anode, and a porous polyethylene separator was placed between the anode and cathode. An electrolyte was added to complete the fabrication of a coin‐type half‐cell battery. The manufactured coin half‐cells were charged (0.2 C) to 4.25 or 4.175 V using the CC–CV method with an end current of 0.05 C at 25 °C, and discharged (0.2 C) using the CC method to 2.5 V for evaluation.

### Cell Preparation and Electrochemical Tests—Cylindrical Cells

The 21700 cylindrical cells were assembled following a conventional method^[^ ^61^ ^]^ using a cathode active material of large‐scale grade and graphite as the anode active material. To optimize cell performance, the loading amount and N/P ratio of each active material, conductive agent content, binder content, separator thickness, and electrolyte composition and content were considered during the design. The assembly process involved preparation of the cathode, separator, and anode according to the design specifications and formation of jelly rolls via a winding process. The resulting jelly rolls were inserted into a cylindrical can and welded to the top and bottom terminals. The cell neck was formed using a well‐known beading process, with a top placed on top of the neck. Thereafter, the electrolyte was injected into the cylindrical cell, followed by welding of the anode tab and top cap to secure them. Finally, crimping and sizing were performed to produce cells with standardized dimensions. To evaluate the capacities of the 21700 cylindrical cells, two continuous cycles were performed. The first cycle involved CC–CV charging to 4.2 V with 0.5 C, terminating when I < C/20, followed by CC discharging to 3.0 V with 0.5 C. The second cycle involved CC–CV charging to 4.1 V with 0.5 C, terminating when I < C/20, followed by CC discharging to 3.0 V with 0.5 C. All capacity tests were conducted using a 10 A cycler (PECC05‐10: Wonik PNE) at 40 °C in a stable temperature chamber. The produced 21700 cells were stored for 2 h to reach thermal equilibrium before testing.

### Operando X‐Ray Diffraction

*Operando* XRD measurements were conducted by assembling coin half‐cells with Kapton windows on both caps to allow X‐ray transmission. The fabrication process and components were the same as those described previously, excluding the addition of Kapton windows. The fabricated cells were charged to 4.25 V at 0.1 C using a potentiostat (VSP‐300, BioLogic) while conducting XRD measurements in the transmission mode (Empyrean: Malvern Panalytical). A Mo target (Mo *Kα* with λ = 0.71 Å) was used as the X‐ray source, and a CdTe detector was used for high‐energy diffraction. The measurements were conducted for 3 min per scan in 2θ = 7–30°. The fraction of the H2 phase was determined by single‐peak fitting using a pseudo‐Voigt profile function and calculating the integrated intensity (TOPAS software, Bruker).

## Conflict of Interest

The authors declare no conflict of interest.

## Supporting information



Supporting Information

## Data Availability

The data that support the findings of this study are available in the supplementary material of this article.
