# Supplementary material for: Atomic‐Scale Tailoring of Ni‐Rich Cathodes for the Development of Commercial Li‐Ion Batteries: From Laboratory to Market
Source: Adv Sci (Weinh). 2025 Nov 6;13(5):e15521. doi: 10.1002/advs.202515521 (PMC12850020; doi:10.1002/advs.202515521)
Supplement: Supplementary file 1 — Supporting Information [file ADVS-13-e15521-s001.docx]

Supporting Information

**Atomic-Scale Tailoring of Ni-Rich Cathodes for the Development of Commercial Li-Ion Batteries: From Laboratory to Market**

*Joonhyeon Kang,^*,+^ Sanghyun Lee, ^+^ Young Cheol Choi, Seungjun Myeong, Sinyoung Park, Hyo Seok Kang, Jia Shin, June Woo Lee, Minkyu You, Sun Sik Shin, Yung Jong Lee, Mira Im, Jong Keun Lee, Jongpil Jegal, Kwan Soo Lee, and Cheol-Hee Park^*^*

J. Kang, S. Lee, Y. C. Choi, S. Myeong, S. Park, H. S. Kang, Y. J. Lee, M. Im, J. K. Lee,

J. Jegal, K. S. Lee, C.-H. Park

R&D Campus, Daejeon
LG Energy Solution
188 Moonji-ro, Yusong-gu, Daejon 34122, Republic of Korea

J Shin, J. Woo Lee, Minkyu You, Sun Sik Shin

R&D Campus, Daejeon
LG Chem
188 Moonji-ro, Yusong-gu, Daejon 34122, Republic of Korea

**Table-of-contents:**

Supplementary Note 1–5

Supplementary Figures 1–15

Supplementary Table 1–10

**Note S1: Determination of crystallographic model for Ni-rich NCM**

For simplicity, we first describe the crystal structure of LiNiO_2_ before discussing Ni-rich NCM. An ideal stoichiometric LiNiO_2_ has an *R*$\bar{3}$*m* space group with Li, Ni, and O occupying the Wyckoff positions 3a (0 0 0), 3b (0 0 0.5), and 6c (0 0 z), respectively, in a hexagonal setting. To describe the synthesized samples, we consider six crystal model candidates: stoichiometric [Li]_3a_[Ni]_3b_[O_2_]_6c_ (Model 0), stoichiometric Li/Ni cation mixing [Li_1-x_Ni_x_]_3a_[Ni_1-x_Li_x_]_3b_[O_2_]_6c_ (Model 1), non-stoichiometric Li deficiency [Li_1-x_]_3a_[Ni]_3b_[O_2_]_6c_ (Model 2), non-stoichiometric Ni excess [Li_1-x_Ni_x_]_3a_[Ni]_3b_[O_2_]_6c_ (Model 3), non-stoichiometric Li excess [Li]_3a_[Ni_1-_*_x_*Li*_x_*]_3b_[O_2_]_6c_ (Model 4), and non-stoichiometric O deficiency [Li]_3a_[Ni]_3b_[O_2-x_]_6c_ (Model 5). Various types of defects (x) can affect the Li/M ratio and Ni oxidation state in different ways, depending on the crystal model (**Figure S5** and **Table S2**). Additionally, the Ni oxidation state changes the occupancy of the 3*d* *t*_2g_ and *e*_g_ orbitals under cubic crystal field splitting and alters the number of unpaired electrons and spin *S*. Furthermore, the magnetic exchange interaction between the in-plane and out-of-plane triangular lattices is altered (**Figure S7**). Therefore, based on structural and magnetic analyses of the synthesized samples, we identify one of the possible crystallographic models.

When the Li/M input ratio does not affect the oxidation state of Ni, it results in stoichiometric LiNiO_2_ and impurity phases caused by excess Li (Ni deficiency) or Ni (Li deficiency). Changes in the Li/M input ratio only cause a variation in the overall Li/M ratio, whereas the unit cell parameters of LiNiO_2_ determined by XRD measurements are expected to remain constant. However, as shown in **Figure 3**a, the *a*- and *c*-lattice parameters, *c*/*a* ratio, and *V* changes linearly depend on the Li/M input ratio. Thus, changing the Li/M ratio can affect the atomic composition of the LiNiO_2_ unit cell. Therefore, we reject the possibility of an ideal stoichiometric [Li]_3a_[Ni]_3b_[O_2_]_6c_ model that maintains its crystal structure while generating impurities.

First, among the remaining five crystal structure candidates, we rule out the Li deficiency model (Model 2: [Li_1-x_]_3a_[Ni]_3b_[O_2_]_6c_). Increasing the Li/M ratio to fill Li in the Li-deficient 3a site induces Ni reduction and increases the number of unpaired electrons, thereby increasing the effective magnetic moment. Contrarily, our results exhibit a trend opposite to that of the Li-deficiency model, as observed from the SQUID measurements, where an increase in the Li/M ratio causes a decrease in the effective magnetic moment (Figure 3e). Second, a consistent Li/M ratio should be maintained within the LiNiO_2_ unit cell to afford a valid oxygen deficiency model, which contradicts the variations in the lattice constants obtained from XRD and can be excluded, similar to Model 0.

Next, we consider the [Li_1-x_Ni_x_]_3a_[Ni_1-y_Li_y_]_3b_[O_2_]_6c_ formula, which includes Li/Ni cation mixing and excess Li or Ni (Models 1, 3, and 4). Based on the Rietveld results, the laboratory-scale samples exhibit distinctive features of finite Ni_Li_ defects and negligible levels of Li_Ni_ defects (~0). Therefore, the simplified Model 3 (non-stoichiometric Ni excess [Li_1-x_Ni_x_]_3a_[Ni]_3b_[O_2_]_6c_) is selected as the model for describing the samples under our synthetic conditions.

**Note S2: Magnetic behavior of LiNiO_2_**

In the [Li_1-x_Ni_x_]_3a_[Ni]_3b_[O_2_]_6c_ structure, different Ni oxidation states (Ni^2+^ at 3a and Ni^3-x^ at 3b) result in unequal spin sizes. We did not observe any evidence of long-range magnetic order until cooling to 5 K in the zero-field cooling magnetic susceptibility measurements at 1 T. When the Li/M ratio is increased, the magnetic susceptibility slowly increases at low temperatures. In the inset of Figure 3d, the inverse magnetic susceptibility curve shifts to the −x direction with increasing the relative Li/M ratio. The Curie–Weiss fitting of the inverse magnetic susceptibility provides the effective magnetic moment and Curie–Weiss temperature, as shown in Figure 3e. As the inverse magnetic susceptibility shifts in the x-direction, the Curie–Weiss temperature decreases with an increase in the Li/M input ratio.

The magnetic behavior of intrinsic LiNiO_2_ depends on the interplay between orbital ordering and magnetic order via octahedral crystal field splitting and electron occupation of Ni^3+^ 3*d* *t*_2g_^6^*e*_g_^1^. At low temperatures, the Jahn–Teller distortion is preferred to lower energy by breaking orbital degeneracy, which induces further crystal symmetry reduction in the monoclinic structure.^1^ However, the average or static crystal structure maintains a rhombohedral *R*$\bar{3}$*m* symmetry with *e*_g_^1^ orbital degeneracy, as observed experimentally. The ferromagnetic *J*_op,FM_ and anti-ferromagnetic *J*_op,AFM_ interactions of the nearest neighbor cooperate with different and similar orbital ordering, respectively.^2^ In such competing interactions, subtle defect-mediated local distortions can have a significant impact on the type of magnetic interactions. When Ni_Li_ defects exist, they induce different oxygen octahedral distortions in neighboring octahedra and different orbital ordering, resulting in strong ferromagnetic spin interactions. Thus, we conclude that the Curie–Weiss temperature is more sensitive to changes in the Li/M ratio than the effective magnetic moment; when the Li/M ratio is altered by 4%, changes of 50 and 1.5% in Curie–Weiss temperature and effective magnetic moment, respectively, are observed (**Figure 3**e).

**Note S3: Equations expressing capacity as a function of Li/M ratio**

For Li_1-z_M_1+z_O_2_ (M = Ni, Co, Mn) with fixed oxidation states of +1 for Li, +3 for Co, +4 for Mn, and −2 for O, and defined oxidation state of Ni as *Ni_ox_*, the following equation is derived to satisfy charge neutrality when the ratio of Ni, Co, and Mn is α:β:γ (α+β+γ=1).

$$\left( 1-z \right)\left( +1 \right)+\left( 1+z \right)\left( \alpha\left( {Ni}_{ox} \right)+\beta\left( +3 \right)+\gamma\left( +4 \right) \right)+2(-2)=0$$

Then, we obtain *Ni_ox_* as

$${Ni}_{ox}={(\frac{z+3}{z+1}-3\beta-4\gamma)}/\alpha$$

Assuming that the oxidation states of Ni and Co are constant at a certain potential (*E*), the available charges from Ni and Co can be expressed as

$Available charge from Ni=\left( {Ni}_{ox}\left( E \right)-{Ni}_{ox} \right)(z+1)\alpha$ (*mol*)

$Available charge from Co=\left( {Co}_{ox}\left( E \right)-{Co}_{ox} \right)(z+1)\beta$ (*mol*)

where *Ni_ox_*(*E*) and *Co_ox_*(*E*) represent the oxidation states of Ni and Co attained at potential *E*, respectively, and *Co_ox_* represents the initial oxidation state of Co as +3.

Therefore,

$$Specific charge=\frac{\left( {Ni}_{ox}\left( E \right)-{Ni}_{ox} \right)\left( z+1 \right)\alpha+\left( {Co}_{ox}\left( E \right)-{Co}_{ox} \right)(z+1)\beta}{M.W. \left( from chemical formula \right)} ({mol\cdot g}^{-1})$$

$$=\frac{\left. \left( {Ni}_{ox}\left( E \right)-\left. (\left. \frac{z+3}{z+1}-3\beta-4\gamma) \right. \right./\alpha\right. \right)\left( z+1 \right)\alpha+\left( {Co}_{ox}\left( E \right)-3 \right)(z+1)\beta}{6.941\left( 1-z \right)+\left( 58.693\alpha+58.933\beta+54.938\gamma\right)\left( z+1 \right)+15.999(2)} \left( mol\cdot g^{-1} \right)$$

The atomic weights of Li, Ni, Co, Mn, and O are 6.941, 58.693, 58.933, 54.938, and 15.999 g, respectively.

From $x=\left( 1-z \right)\left( 1+z \right) \leftrightarrow z=(1-x)(1+x)$,

$$Specific charge=\frac{C_{1}-2x}{C_{2}+45.88x} (mol\cdot g^{-1})$$

where$C_{1}=2\alpha\times{Ni}_{ox}\left( E \right)+2\beta\times{Co}_{ox}\left( E \right)+8\gamma-4$ (constant at potential *E*)

$$C_{2}=2(58.693\alpha+58.933\beta+54.938\gamma+15.999)$$

Additionally,

$$Specific capacity=Specific charge \left( {mol\cdot g}^{-1} \right)\times96485 ({C\cdot mol}^{-1})\times1000/3600 ({mAh\cdot C}^{-1})$$

**Note S4: Bond Valence Sum (BVS) calculation**

We calculated the lattice parameters and the Ni occupancy x at the Wyckoff 3a site based on the [Li_1-x_Ni_x_]_3a_[Ni]_3b_[O_2_]_6c_ ​model using the Rietveld method. A linear fit was then applied to the data in the range of x=0 to x=0.02 to determine the evolution of the lattice constants, as shown in the figure below (**Figure S1**).


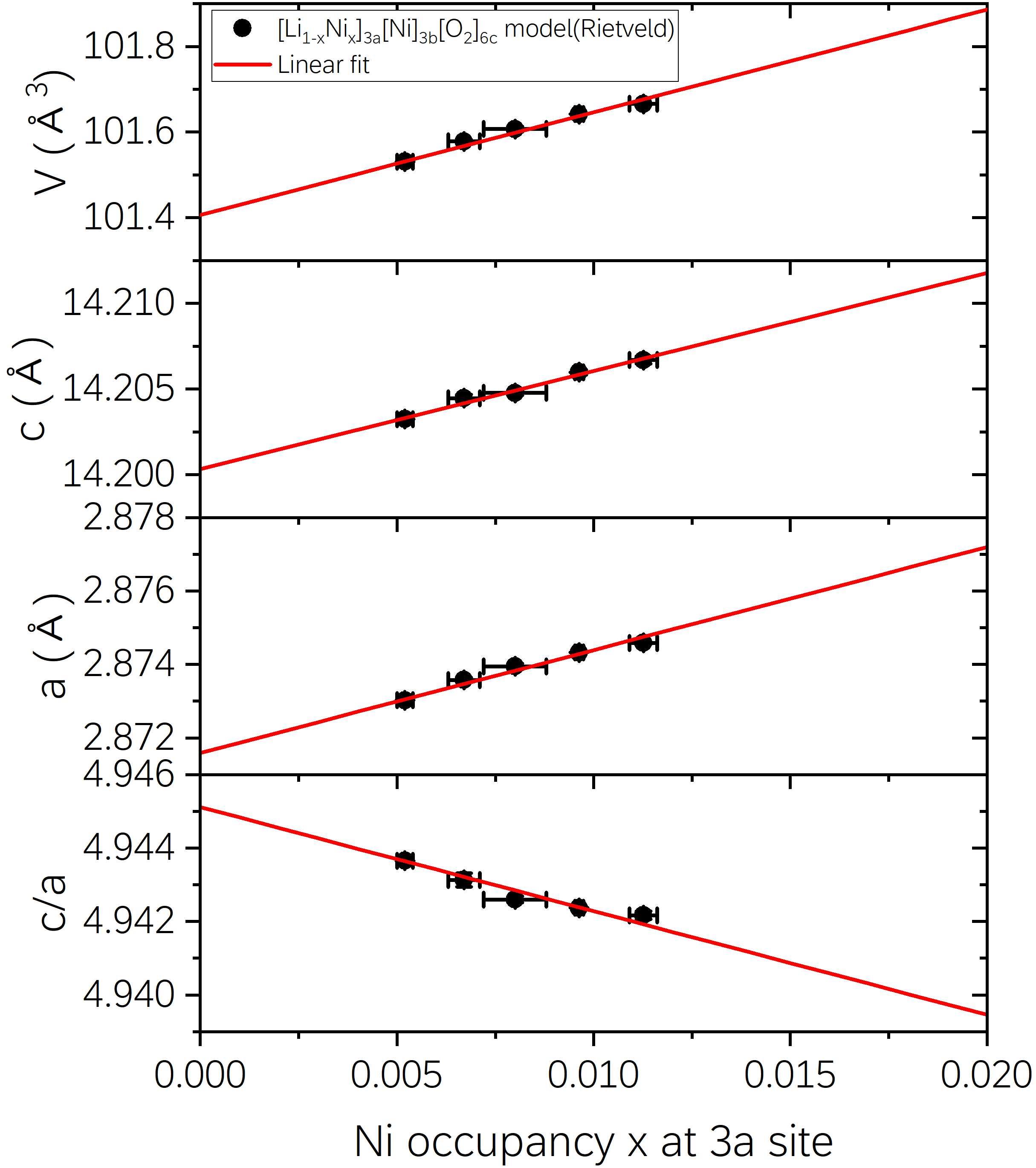


**Figure S1**. Lattice parameters (a, c), c/a ratio, and unit cell volume as a function of Ni occupancy (x) at the Wyckoff 3a site (0, 0, 0).

With the oxygen position fixed at (0 0 z) with z=0.2407±0.0001, we performed Bond Valence Sum (BVS) calculations to obtain the charge of each atom (**Figure S12**). The charge Q is defined as: Q=$\sum_{i} n_{i}s_{i}$ where *n*_i_ is the number of bonds, and *s*_i_ is given by *s*_i_=exp{(*d*_0_-*d*)/B_0_}, with *d* being the bond length. For the Li–O, Ni(3a site)–O, and Ni(3b site)–O bonds, we used *d*_0_=1.466, 1.654, and 1.686, respectively, with B_0_=0.37. The parameters were obtained from the BondStr program in the Fullprof suite.

Accordingly,

- Q_Li_=$\sum_{i} n_{i}s_{i}$=6$*s_{Li-O}$
- Q_Ni(3a)_=$\sum_{i} n_{i}s_{i}$=6*$s_{Ni(3a)-O}$
- Q_Ni(3b)_=$\sum_{i} n_{i}s_{i}$=6*$s_{Ni(3b)-O}$
- Q_O_=$\sum_{i} n_{i}s_{i}$=(3-x)*$s_{Li-O}$+x*$s_{Ni(3a)-O}$+3*$s_{Ni(3b)-O}$

**Note S5: Additional DFT calculations**

We calculated the density of states (DOS) using the GGA+U method for models with Ni_Li_ defects (LiNiO₂ w/ Ni_Li_) and for the defect-free model (bare LiNiO₂), in the fully lithiated (**Figure S2a**) and delithiated states (**Figure S2b**). As shown in the figure, for the LiNiO₂ model containing Ni_Li_ defects, residual electrons remain in the *e_g_* state in the delithiated H3 phase. This indicates the presence of Ni³⁺ and suggests instability arising from Jahn–Teller distortion, which may contribute to the relatively higher V_H2–H3_ observed near the end of charging. In contrast, for the bare LiNiO₂ model, no DOS is observed near the Fermi level and a band gap opens, indicating a highly uniform and stable state without Jahn–Teller distortion. This stabilization could explain the relatively lower H2–H3 transition voltage in this case.


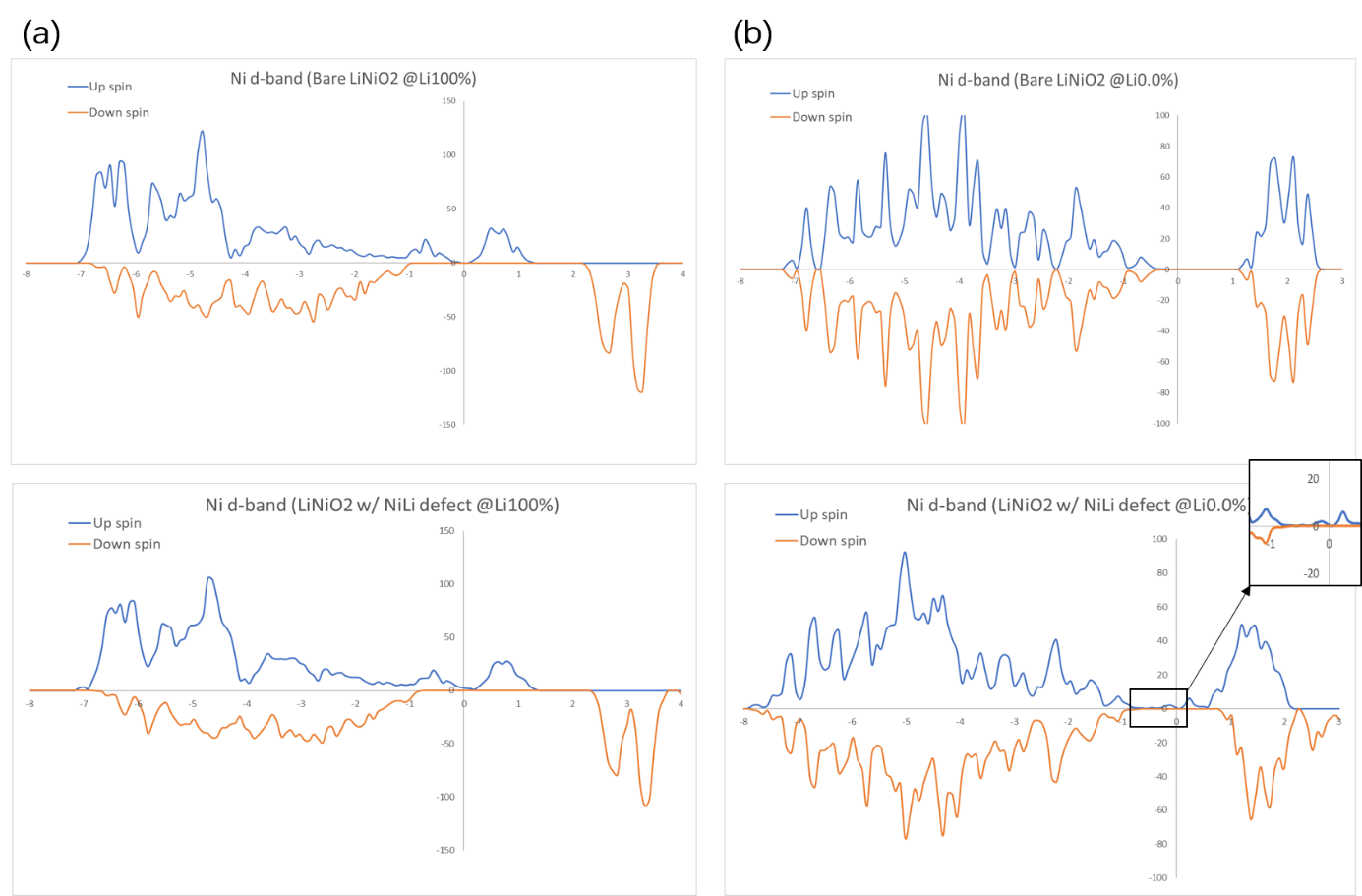


**Figure S2**. Density of states (DOS) calculations for (a) the fully lithiated and (b) the delithiated states in models with Ni_Li_ defects (LiNiO₂ w/ Ni_Li_) and in the defect-free model (bare LiNiO₂).


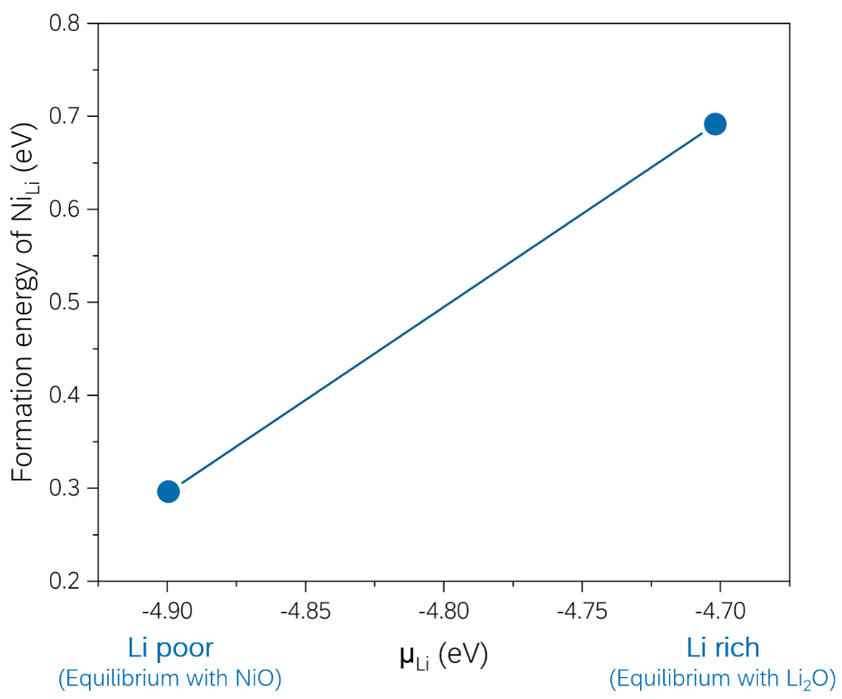


**Figure S3.** Formation energy of the Ni_Li_ defect in a Li-poor environment (equilibrium with NiO) and Li-rich environment (equilibrium with Li_2_O) of LiNiO_2_. An increase in the Li/M ratio creates a relatively Li-rich environment during synthesis. The values used for the calculations are specified in Table S6.


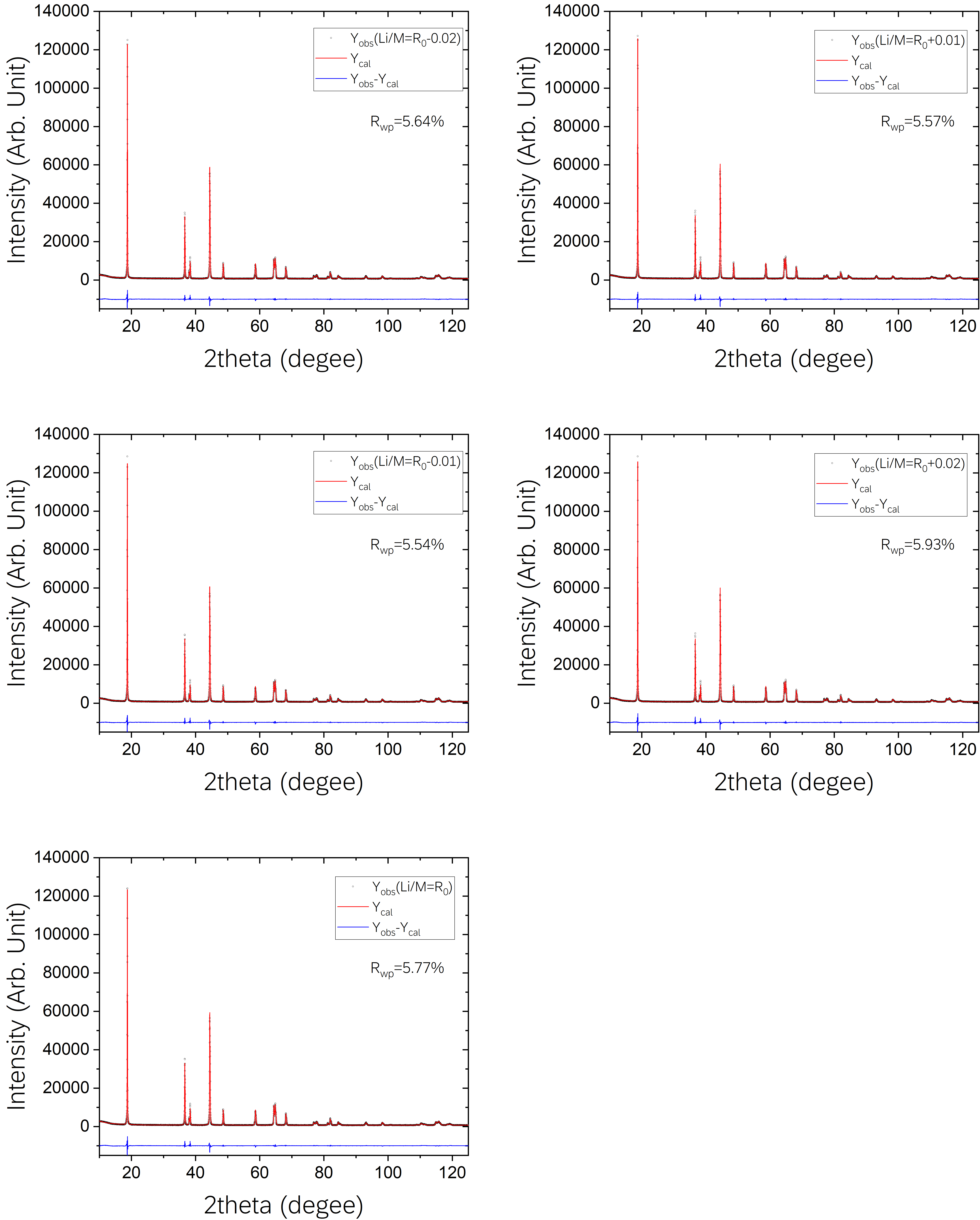


**Figure** **S4.** Representative powder XRD patterns and Rietveld refinement results.


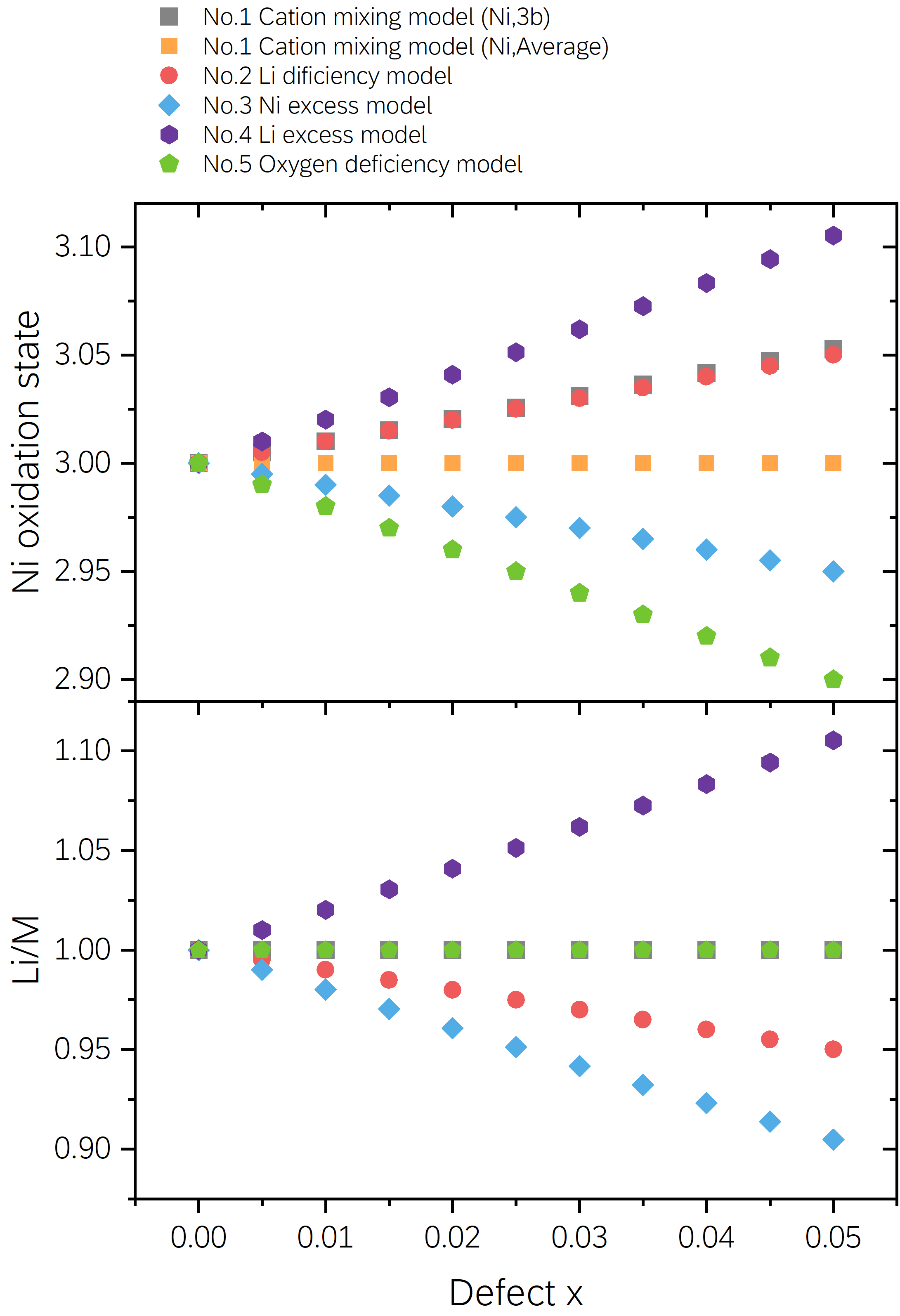


Figure S5. Variations in Li/M and Ni oxidation states within each crystal model calculated from the types and concentrations of defects (Table S2).


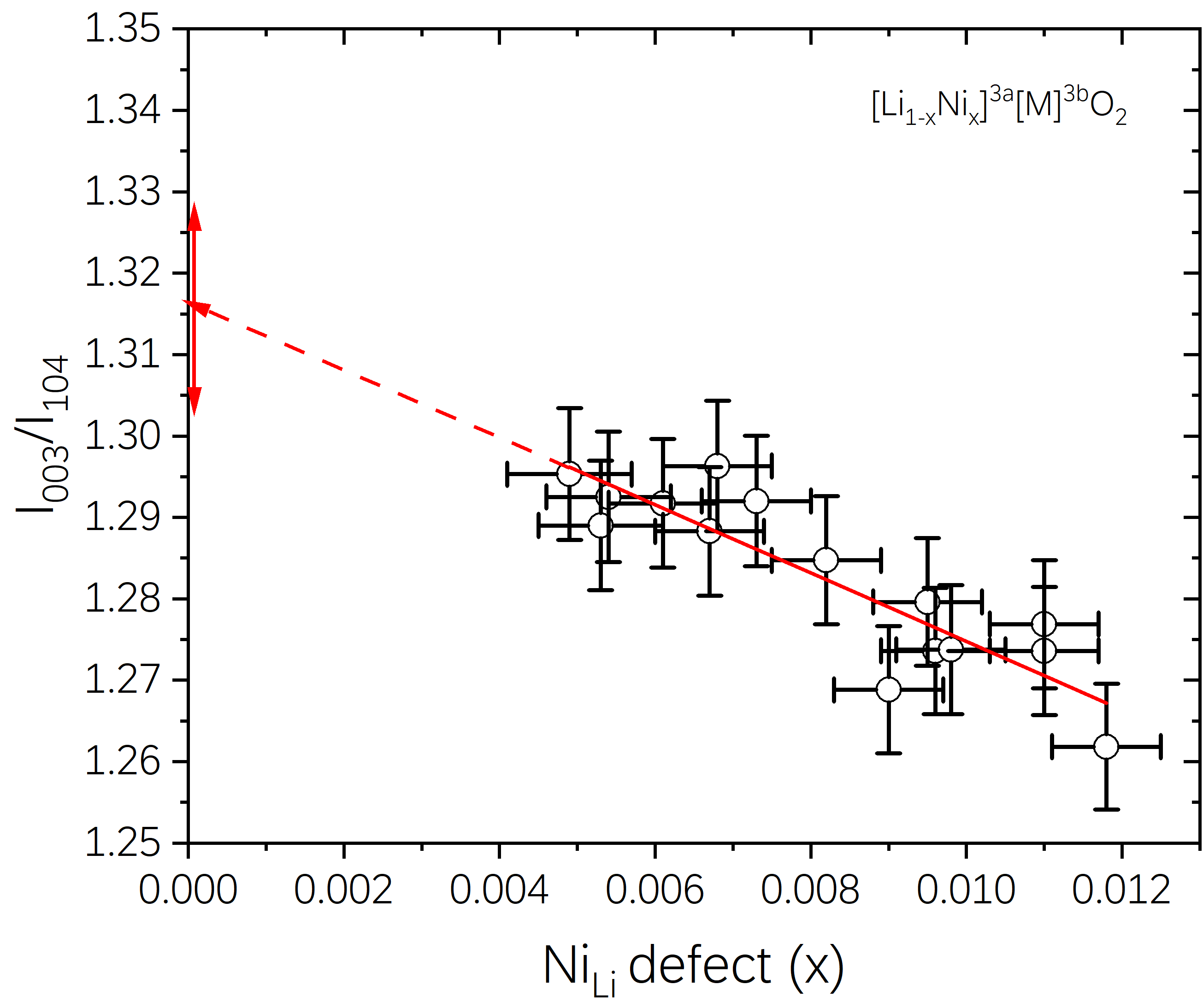


**Figure** **S6.** Plot of the I_003_/I_104_ ratio versus Ni_Li_ defect concentration in Ni-rich NCM (LiMO_2_). The ideal stoichiometric Ni-rich NCM is expected to have an I_003_/I_104_ ratio of 1.315(10).


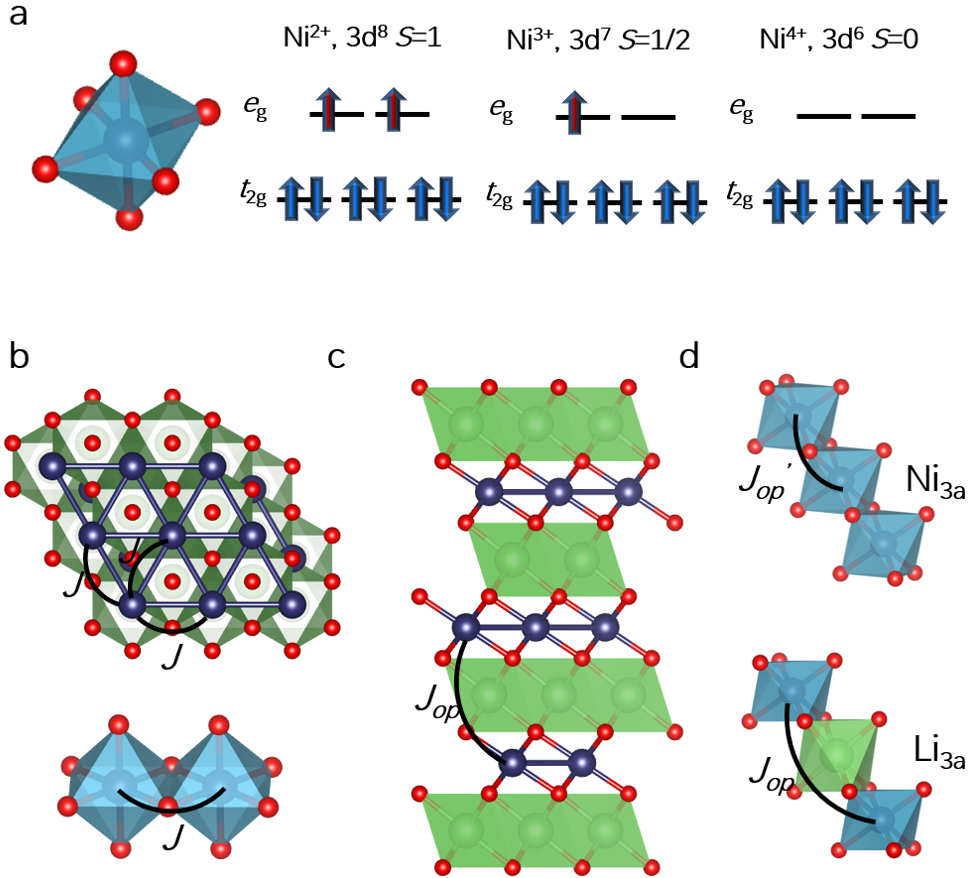


Figure S7. Spin states under an octahedral crystal field and magnetic interaction pathway. a. Ni spin states of Ni^2+^, Ni^3+^, and Ni^4+^ under an octahedral crystal field splitting. b. Ni triangular lattice of an edge-sharing NiO_6_ octahedral with an in-plane exchange interaction *J* c. Out-of-plane exchange interaction *J_op_* between triangular lattice layers. d. Exchange interaction when Ni occupies the Li 3a site. Ni_Li_ generates an additional exchange interaction *J_op_*' between the triangular lattice layers.


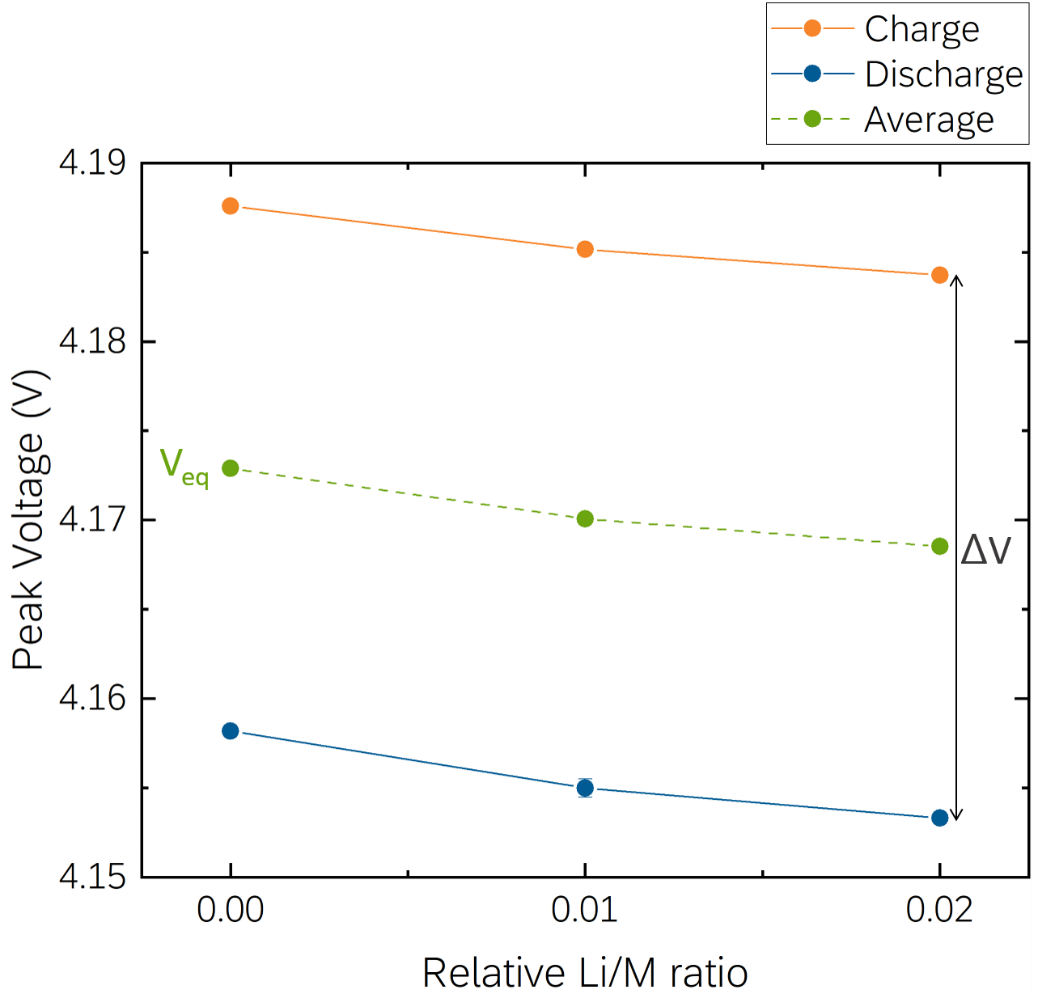


**Figure S8.** Variation of the charge and discharge dQ/dV peak voltages corresponding to the H2–H3 transition as a function of the Li/M ratio, obtained from Gaussian fitting, together with the derived average (equilibrium) voltage (V_eq_) and voltage hysteresis (ΔV). With increasing Li/M ratio, both charge and discharge dQ/dV peak voltages shift toward lower values, while ΔV remains nearly constant at ~0.030 V.


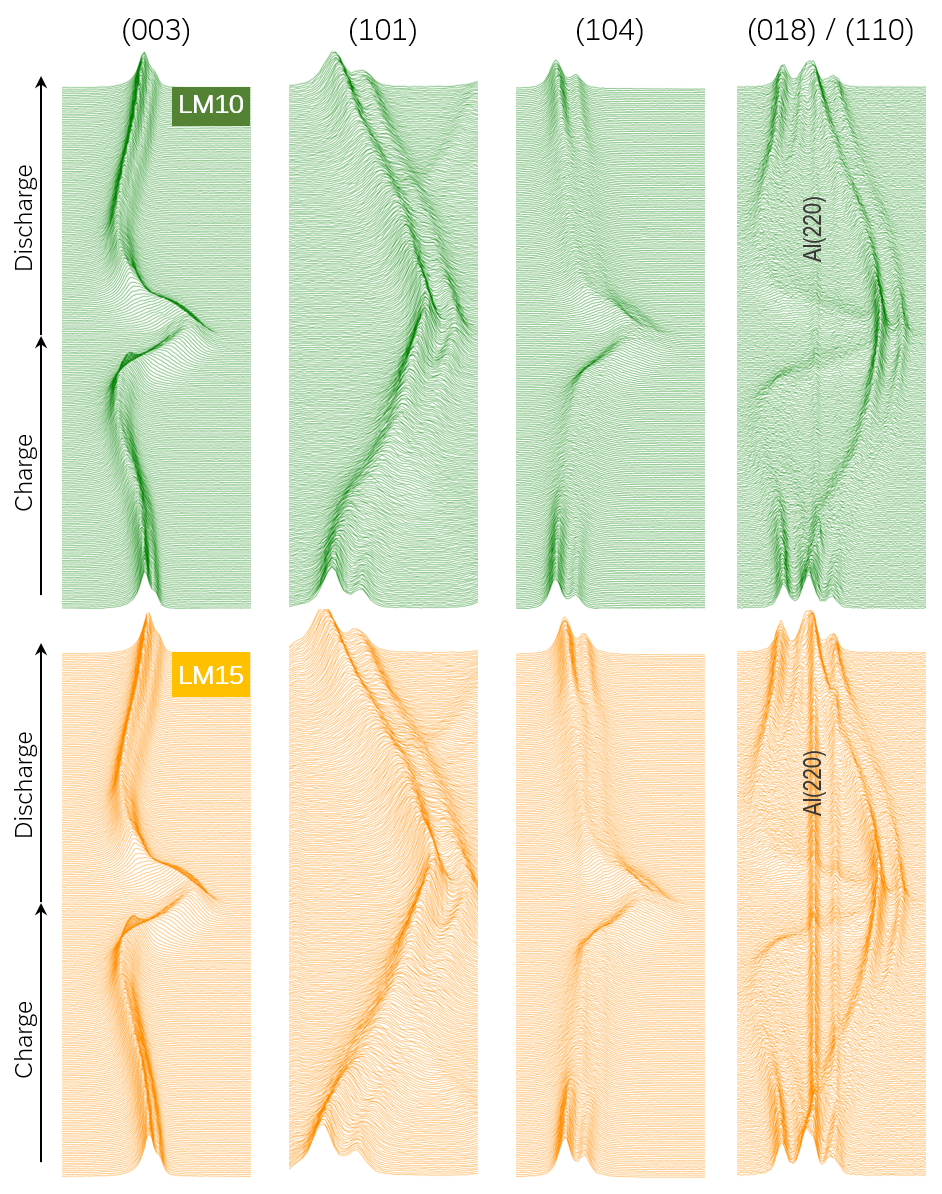


**Figure S9.** Operando XRD patterns collected during charge–discharge cycling of LM10 and LM15.


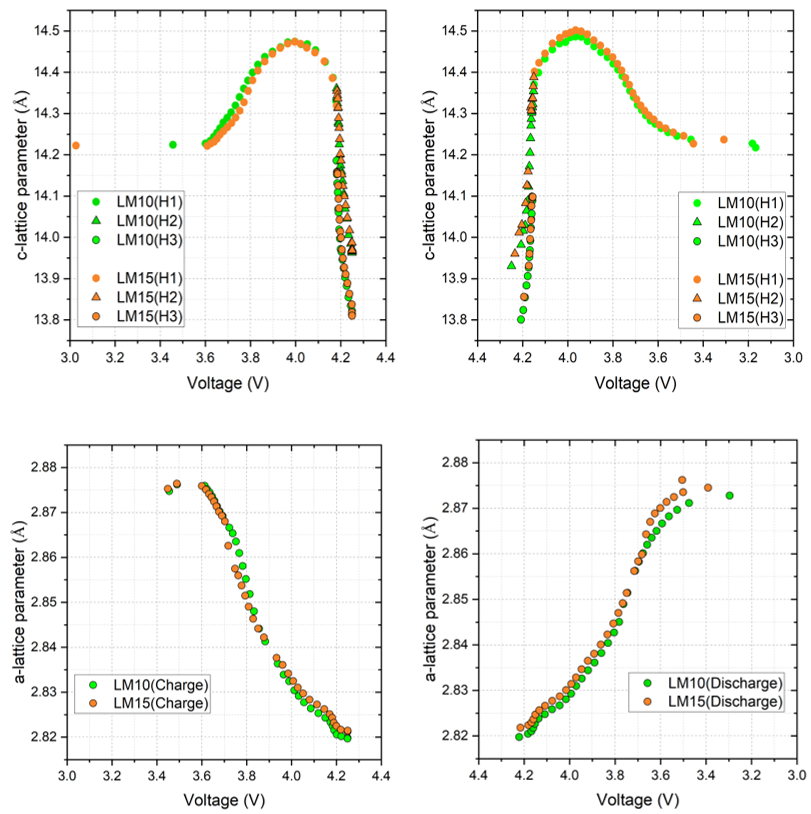


**Figure S10.** Evolution of the (a) c- and (b) a-lattice parameters of LM10 and LM15 during charge–discharge cycling. The c-lattice parameter was determined from two-phase fitting of the (003) reflection to follow the H2–H3 phase transition, whereas the a-lattice parameter was obtained from single-phase fitting of the (110) reflection, as detailed in Figure S9.


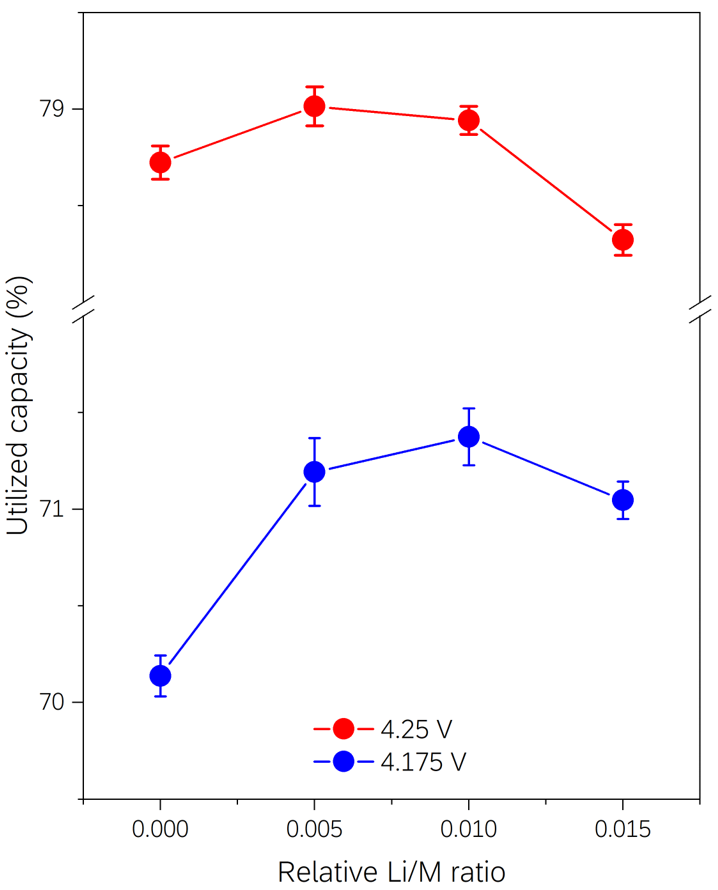


**Figure** **S11.** Discharge capacity after charging up to cut-off voltages of 4.175 and 4.25 V, represented as a ratio of utilized to theoretical capacities of stoichiometric Ni-rich NCM.


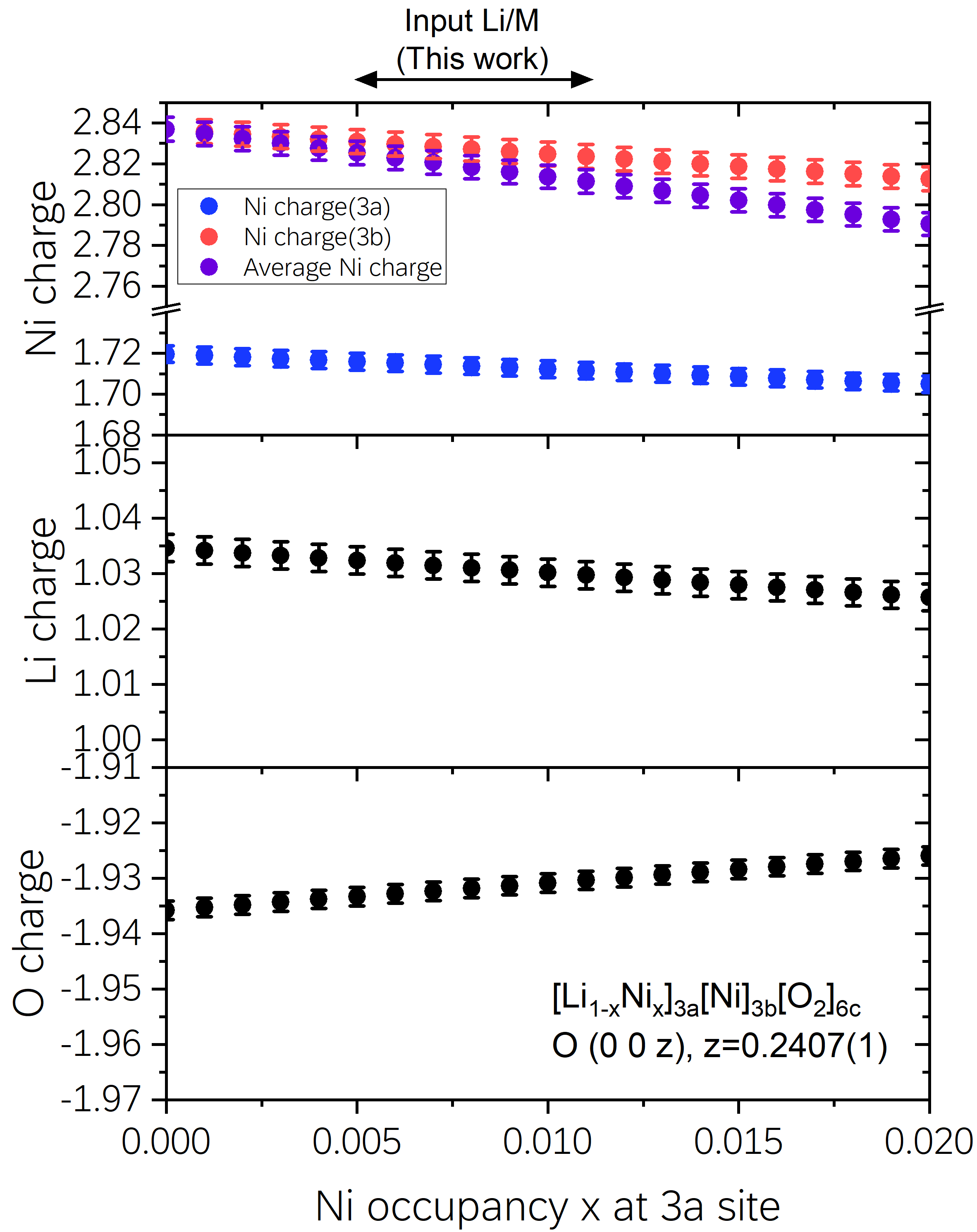


**Figure S12.** Results of Bond Valence Sum (BVS) calculations based on the [Li_1−x​_Ni_x_]_3a​_[Ni]_3b_​[O_2_​]_6c_​ structural model. BVS-derived charges of Li, Ni at the 3a and 3b sites, and O at the 6c site with z=0.2407(1). The results indicate that a decrease in Ni occupancy at the 3a site leads to increased Ni oxidation at the 3b site, accompanied by correlated changes in pillar stability and Ni redox activity.


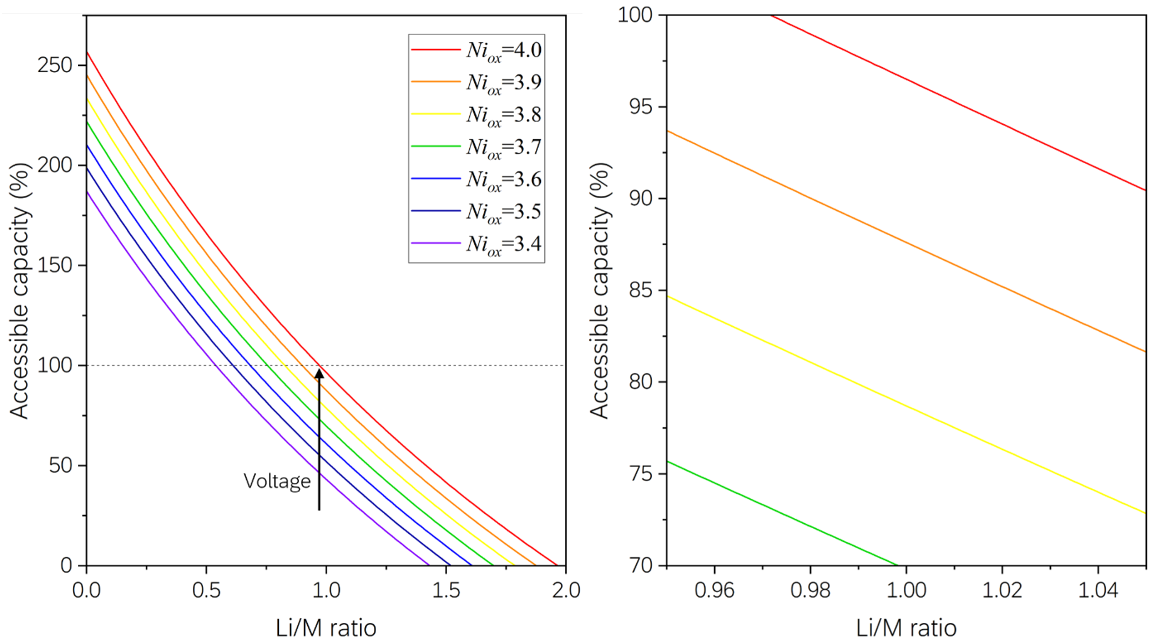


**Figure** **S13.** Changes in the capacity resulting from variations in the Li/M ratio, calculated without considering constraints on the available Li quantity based on the previously derived equation (Note S3). Several plots that simulate an increase in the oxidation state of Ni from 3.4 to 4.0 at regular intervals of 0.1 are presented, while maintaining the oxidation state of Co at 3.5, reflecting a situation where the oxidation state of Ni increases upon charging. Within the compositional range of interest, as shown in the graph on the right that enlarges the area of interest, the capacity values approximate a linear relationship, with the slope remaining nearly constant regardless of the Ni oxidation state. The capacity values are expressed as relative ratios to the theoretical capacity of the stoichiometric composition.


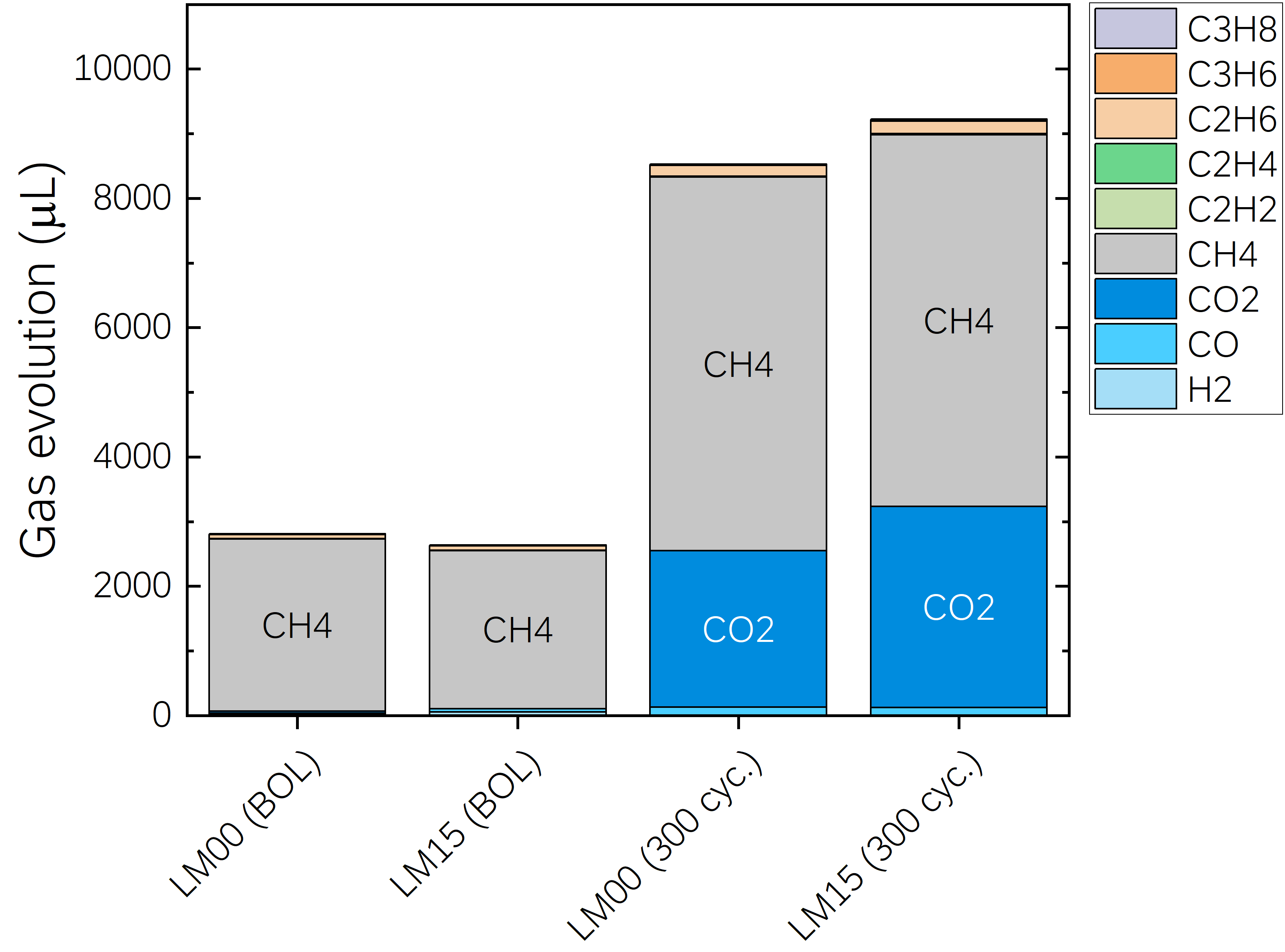


**Figure S14**. Gas evolution behavior of LM00 and LM15 commercial cylindrical cells. Gas evolution was analyzed at the beginning of life (after cell activation) and after 300 cycles under long-term cycling conditions (0.5 C charge to 4.1 V with CV hold at C/20, rest 10 min, discharge to 3.0 V, 40 °C). Measurements were performed in the discharged state using gas collection and analysis. Both LM00 and LM15 exhibit comparable levels of gas generation.


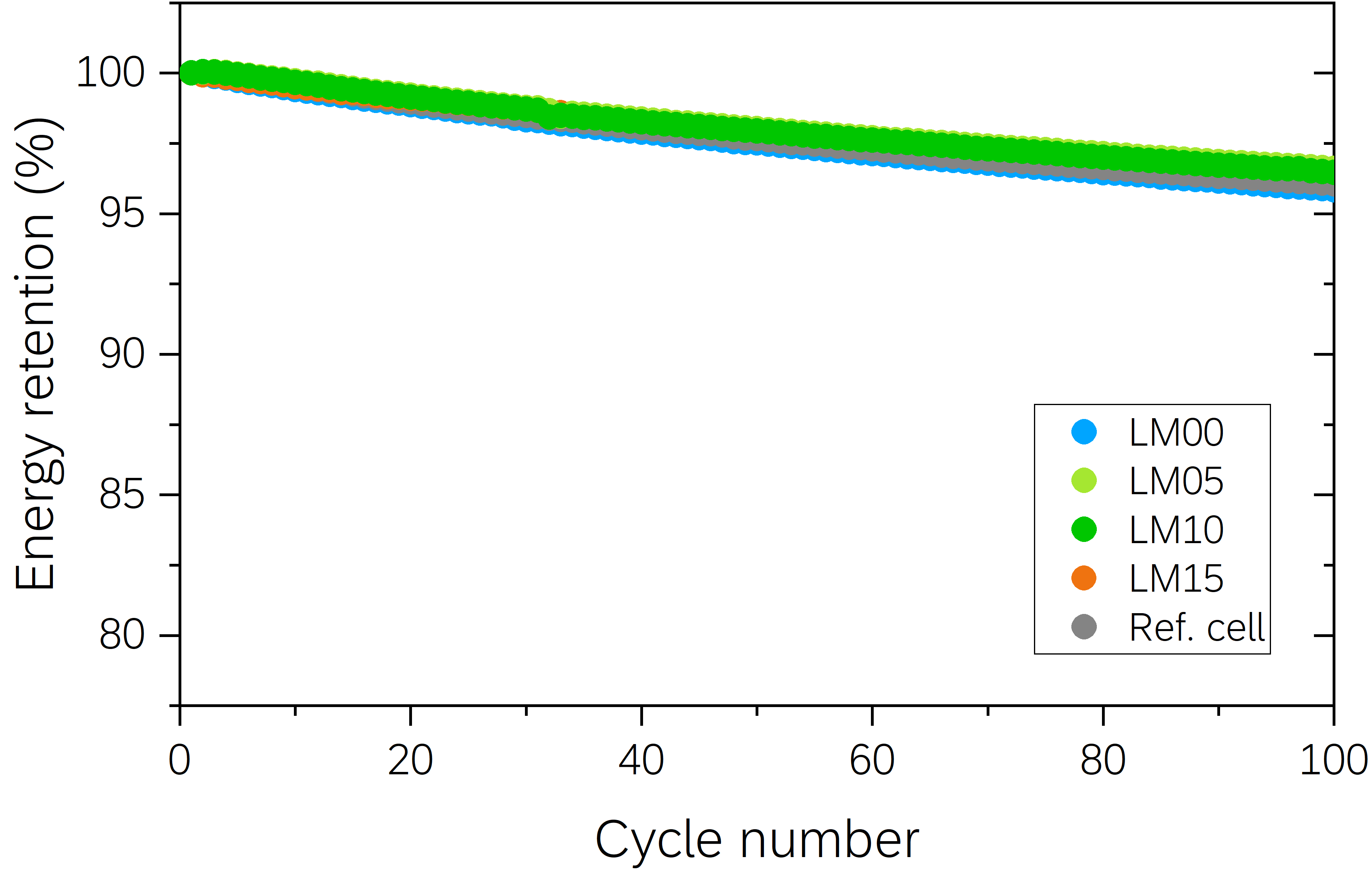


**Figure** **S15.** The energy retention during the operation of cylindrical cells manufactured with our mass-produced grades, at a 0.3 C, within the voltage range of 2.85 to 4.2 V, over 100 cycles at 40°C. For comparison, cylindrical cells manufactured simultaneously with our reference NCM were also evaluated during their respective cycling.

**Table** **S1.** Detailed XRD Rietveld refinement results of powder samples with controlled Li/M ratio. We performed Rietveld refinement using the crystal structure model of [Li_1-_*_x_*Ni*_x_*]_3a_[Ni_1-_*_y_*Li*_y_*]_3b_[O_2_]_6c_, which can consider all models 1, 3, and 4, under the initial conditions of x≠0, y≠0, and x≠y. When x=y, it corresponds to the stoichiometric Li/Ni cation mixing [Li_1-_*_x_*Ni*_x_*]_3a_[Ni_1-_*_x_*Li*_x_*]_3b_[O_2_]_6c_ (Model 1), when *y*=0, it corresponds to the non-stoichiometric Ni excess [Li_1-_*_x_*Ni*_x_*]_3a_[Ni]_3b_[O_2_]_6c_ (Model 3), and when x=0, it corresponds to the non-stoichiometric Li excess [Li]_3a_[Ni_1-_*_y_*Li*_y_*]_3b_[O_2_]_6c_ (Model 4).

| *R*-3*m* (Space group. No. 166, hexagonal setting)  Wyckoff position 3a (0 0 0), 3b (0 0 0.5), 6c (0 0 z) | | | | | |
| --- | --- | --- | --- | --- | --- |
| Relative Li/M | -0.02 | -0.01 | 0 | 0.01 | 0.02 |
| *V* (Å^3^) | 101.666(3) | 101.642(2) | 101.607(6) | 101.578(4) | 101.531(1) |
| *a* (Å) | 2.87459(3) | 2.87432(1) | 2.87395(8) | 2.87358(7) | 2.87303(2) |
| *c* (Å) | 14.2067(2) | 14.2060(2) | 14.2048(1) | 14.2045(2) | 14.2033(2) |
| O_z_ (0 0 z) | 0.2407(1) | 0.2408(1) | 0.2407(1) | 0.2408(1) | 0.2408(1) |
| *x*  ([Li_1-x_Ni_x_]_3a_) | 0.0113(4) | 0.0096(1) | 0.0080(8) | 0.0067(4) | 0.0052(2) |
| *y*  ([Ni_1-y_Li_y_]_3b_) | ~0 | ~0 | ~0 | ~0 | ~0 |
| *B_iso,over_* (Å^2^) | 0.48(1) | 0.48(1) | 0.47(1) | 0.49(1) | 0.49(2) |
| Crystalline size (nm) | 133(3) | 127(9) | 131(2) | 127(5) | 124(1) |
| Microstrain (%) | 0.0494(1) | 0.0469(6) | 0.0471(3) | 0.0467(4) | 0.0454(5) |
| *R*_wp_ (%) | 5.50 | 5.55 | 5.62 | 5.61 | 6.06 |

**Table** **S2.** Ni oxidation state calculation for each crystal model considering a simple assumption that Li (at the 3a and 3b sites), the Ni ion (at the 3a site), and O (at the 6c site) have fixed oxidation states of +1, +2, and −2 respectively. By definition, the defect x in each model has a positive value.

| **No.** | **Crystal model** | **Chemical formula** | **Li/M ratio** | **Ni oxidation state Q_Ni_** |
| --- | --- | --- | --- | --- |
| **0** | **Stoichiometric LiNiO_2_**  **[Li]_3a_[Ni]_3b_[O_2_]_6c_** | **LiNiO_2_** | **1** | **Q_Li_+Q_Ni_+2Q_O_=0 (Charge neutrality)**  **Q_Li_=+1, Q_O_=-2, Q_Ni_=+3** |
| **1** | **Stoichiometric Li/Ni cation mixing**  **[Li_1-x_Ni_x_]_3a_[Ni_1-x_Li_x_]_3b_[O_2_]_6c_** | **LiNiO_2_** | **1** | **Q_Li,3a_=+1, Q_Ni,3a_=+2, Q_Li,3b_=+1, Q_O,6c_=-2**  **(+1)(1-x)+(+2)(x)+(Q_Ni,3b_)(1-x)+(+1)(x)+(-2)(2)=0**  **Q_Ni,3b_=(3-2x)/(1-x), Q_Ni,Average_=3** |
| **2** | **Non-stoichiometric Li deficiency**  **[Li_1-x_]_3a_[Ni]_3b_[O_2_]_6c_** | **Li_1-x_NiO_2_** | **1-x** | **Q_Li,3a_=+1, Q_O,6c_=-2**  **(+1)(1-x)+(Q_Ni, 3b_)(1)+(-2)(2)=0**  **Q_Ni,3b_=3+x** |
| **3** | **Non-Stoichiometric Ni excess**  **[Li_1-x_Ni_x_]_3a_[Ni]_3b_[O_2_]_6c_** | **Li_1-x_Ni_1+x_O_2_** | **(1-x)/(1+x)** | **(+1)(1-x)+(+2)(x)+(Q_Ni,3b_)(1)+(-2)(2)=0**  **Q_Ni,3b_=3-x** |
| **4** | **Non-Stoichiometric Li excess**  **[Li]_3a_[Ni_1-x_Li_x_]_3b_[O_2_]_6c_** | **Li_1+x_Ni_1-x_O_2_** | **(1+x)/(1-x)** | **(+1)(1)+(Q_Ni,3b_)(1-x)+(+1)(x)+(-2)(2)=0**  **Q_Ni,3b_=(3-x)/(1-x)** |
| **5** | **Non-Stoichiometric O deficiency**  **[Li]_3a_[Ni]_3b_[O_2-x_]_6c_** | **LiNiO_2-x_** | **1** | **(+1)(1)+(Q_Ni,3b_)(1)+(-2)(2-x)=0**  **Q_Ni,3b_=3-2x** |

**Table S3.** Lattice parameters of perfect LiNiO₂ and LiNiO₂ models containing Ni_Li_, as obtained from DFT calculations. An increase in the Li/M ratio reduces the number of Ni_Li_ defects, resulting in decreased a and c lattice parameters.

| **System** | | **Lattice Parameter (Å)** | |
| --- | --- | --- | --- |
|  |  | ***a*** | ***c*** |
| LiNiO_2_ | w/ Ni_Li_ | 2.910 | 14.247 |
|  | Perfect | 2.908 | 14.237 |

**Table** **S4.** Quantitative analysis of the H2–H3 transition as a function of the Li/M ratio. Charge and discharge dQ/dV peak voltages were obtained from Gaussian fitting of the dQ/dV curves. The average (equilibrium) voltage (V_eq_) and voltage hysteresis (ΔV) were calculated as the mean and difference of the fitted charge and discharge peak voltages, respectively. The results show systematic shifts of the peak voltages toward lower values with increasing Li/M ratio, while ΔV remains nearly constant at ~0.030 V.

| **Rel. Li/M** | **Peak Voltage (V)** | | **Average  Voltage (V)** | **Hysteresis ΔV (V)** |
| --- | --- | --- | --- | --- |
|  | **Charge** | **Discharge** |  |  |
| 0 | 4.1876 | 4.1582 | 4.1729 | 0.0294 |
| 0.01 | 4.1852 | 4.1550 | 4.1701 | 0.0302 |
| 0.02 | 4.1837 | 4.1533 | 4.1685 | 0.0304 |

**Table** **S5.** Designed Li/M input ratios and ICP-measured Li/M ratios obtained after synthesis for each grade. An excess of Li content was observed after synthesis, which is attributed to impurities rather than the structural Li/M ratio. In NCM cathode materials, there is always a certain amount of LiOH and Li_2_CO_3_ present [3,4]. Despite undergoing purification processes in all cathode materials, they still contain impurities such as the ones mentioned above, including moisture. However, these impurities persist without any noticeable trend with respect to Li/M variations. Considering these impurities, when corrected, the structural Li/M ratio in the cathode materials may drop below 1.

| **Grade** | **Li/M input ratio (Designed)** | **Measured Li/M ratio (After synthesis)** |
| --- | --- | --- |
| LM00 | R_0_ | 1.019 |
| LM05 | R_0_+0.005 | 1.025 |
| LM10 | R_0_+0.01 | 1.025 |
| LM15 | R_0_+0.015 | 1.030 |

**Table** **S6.** Total energies obtained through DFT calculations, with an atomic ratio of 1/48 for Ni_Li_. To determine the oxygen chemical potential (µ_O_), we use half of the total energy of an O_2_ molecule in a large cubic cell with a lattice parameter of 15 Å, assuming an oxygen-rich condition. The formation energies of the Ni_Li_ defects are then calculated under both Li-rich (equilibrium of LiNiO_2_ with Li_2_O) and Li-poor conditions (equilibrium of LiNiO_2_ with NiO).

| **System** | | **E_total_ (eV/unit)** | **µ_O_ (eV)** | **µ_Li_ (eV)** | **µ_Ni_ (eV)** | **Formation E**  **of Ni_Li_ (eV)** |
| --- | --- | --- | --- | --- | --- | --- |
| LiNiO_2_ | Perfect | −19.460 | - | - | - | - |
|  | w/ Ni_Li_ | −19.449 | - | - | - | - |
| O_2_ | | −9.886 | −4.943 | - | - | - |
| Li_2_O | | −14.347 | −4.943 | −4.702 | −4.872 | 0.691 |
| NiO | | −9.617 | −4.943 | −4.900 | −4.674 | 0.296 |

**Table** **S7.** Long-term cycling stability of LM00 and LM15 commercial cylindrical cells. Tests were performed at 40 °C with 0.5 C charging (to 4.1 V, CV held until C/20, followed by 10 min rest) and 0.5 C discharging to 3.0 V.

| **Grade** | **Energy Retention (%)** | | | |
| --- | --- | --- | --- | --- |
|  | **100 cycles** | **200 cycles** | **300 cycles** | **500 cycles** |
| LM00 | 95.3 | 92.7 | 90.7 | 88.1 |
| LM15 | 95.6 | 93.6 | 92.1 | 89.6 |

**Table** **S8.** High-temperature storage performance of LM00 and LM15. Cells were stored at 55 °C in the fully charged state (4.2 V). Post-storage capacities were determined by 0.5 C cycling: CC–CV charging to 4.2 V (CV until C/20) followed by discharge to 2.5 V.

| **Grade** | **Energy Retention (%)** | | |
| --- | --- | --- | --- |
|  | **2 mo** | **3 mo** | **6 mo** |
| LM00 | 95.6 | 94.2 | 89.5 |
| LM15 | 96.1 | 95.2 | 90.5 |


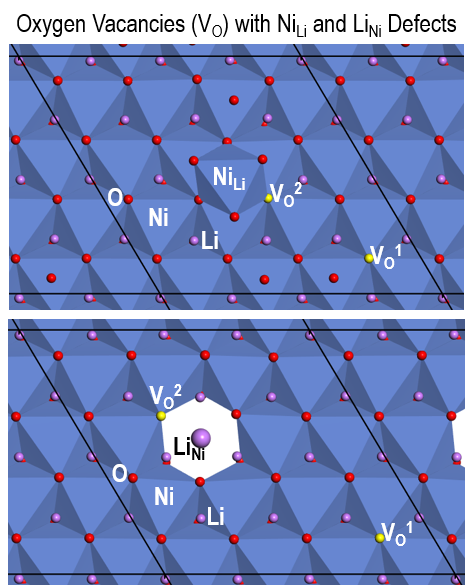
**Table S9.** Formation energies of oxygen vacancies coordinated to defects (V_O_^2^) and oxygen vacancies coordinated to regular lattice sites (V_O_^1^) in models containing Li_Ni_ or Ni_Li_ defects, compared with the formation energy of oxygen vacancies in the perfect model.

| **System** | | ***E_f_*​(*V_O_*​) (eV)** | |
| --- | --- | --- | --- |
|  |  | ***V_O_^2^*** | ***V_O_^1^*** |
| LiNiO_2_ | w/ Ni_Li_ | 1.61 | 1.26 |
|  | Perfect | 1.28 | 1.28 |
|  | w/ Li_Ni_ | 0.87 | 1.25 |

**Table** **S10.** Energy retention after 100 cycles compared to the initial value for cylindrical cells. The evaluation results of our reference NCM, manufactured and cycled simultaneously with each cylindrical cell, are also included.

| **Grade** | **Retention (%)** | **Ref. Cell (%)** |
| --- | --- | --- |
| LM00 | 95.8 | 95.7 |
| LM05 | 96.6 | 95.9 |
| LM10 | 96.5 | 95.9 |
| LM15 | 96.6 | 96.2 |

**Supplementary References**

1. S. Sicolo, M. Mock, M. Bianchini, and K. Albe, And Yet It Moves: LiNiO_2_, a Dynamic Jahn-Teller System, *Chem. Mater.* **32**(23), 10096-10103 (2020)
2. F. Reynaud, D. Mertz and F. Celestini, J.-M. Debierre, A. M. Ghorayeb, P. Simon, A. Stepanov, J. Voiron, and C. Delmas, Orbital Frustration at the Origin of the Magnetic Behavior in LiNiO_2_, *Phys. Rev. Lett.* **86**, 3638 (2001)
3. S. H. Song, S. Hong, M. Cho, J.-G. Yoo, H. M. Jin, S.-H. Lee, M. Avdeev, K. Ikeda, J. Kim, S. C. Nam, S.-H. Yu, I. Park, and H. Kim, Rational design of Li off-stoichiometric Ni-rich layered cathode materials for Li-ion batteries. *Chem. Eng. J.* **448**, 137685 (2022)
4. W. Wang, Y. Shi, P. Li, R. Wang, F. Ye, X. Zhang, W. Li, Z. Wang, C. Xu, D. Xu, Q. Xu, and X. Cui, Rational rock-salt phase engineering of a nickel-rich layered cathode interface for enhanced rate and cycling stability. *Energy* *Environ*. *Sci*. **17** 4283-4294 (2024)
